# Quantifying the impact of air pollution from coal-fired electricity generation on crop productivity in India

**DOI:** 10.1073/pnas.2421679122

**Published:** 2025-02-03

**Authors:** Kirat Singh, David B. Lobell, Inês M. L. Azevedo

**Affiliations:** ^a^Emmett Interdisciplinary Program in Environment and Resources, Stanford University, Stanford, CA 94305; ^b^Department of Earth System Science, Stanford University, Stanford, CA 94305; ^c^Center on Food Security and the Environment, Stanford University, Stanford, CA 94305; ^d^Department of Energy Science and Engineering, Doerr School of Sustainability, Stanford University, Stanford, CA 94305; ^e^Precourt Institute for Energy, Stanford University, Stanford, CA 94305; ^f^Woods Institute for the Environment, Stanford University, Stanford, CA 94305; ^g^Civil and Environmental Engineering (courtesy), Stanford University, Stanford, CA 94305; ^h^Visiting Professor, Nova School of Business and Economics, Carcavelos 2775-405, Portugal

**Keywords:** air pollution, coal electricity, India, food security

## Abstract

Air pollution from coal electricity generation is a major driver of poor air quality in India and its effects on human health have been extensively studied. Despite considerable evidence that the same pollution also reduces crop productivity, we lack similar quantitative assessments of coal electricity’s crop damages. Here, we estimate rice and wheat crop losses from coal generation’s nitrogen dioxide (NO_2_) emissions using a regression model that combines station-level electricity generation and wind direction, satellite-measured NO_2_, and its association with crop productivity. Coal emissions impact yields up to 100 km away from power stations. In parts of West Bengal, Madhya Pradesh, and Uttar Pradesh heavily exposed to coal-linked NO_2_, annual yield losses exceed 10%, equivalent to approximately 6 y worth of average annual yield growth in both rice and wheat in India between 2011 and 2020. While station-specific crop damages (value of lost output) are almost always lower than mortality damages (monetized value of annual premature PM_2.5_-related deaths), crop damage intensity (crop damage per GWh of electricity generated) is frequently higher than mortality damage intensity (mortality damage/GWh). Rice damage intensity exceeds mortality damage intensity at 58, and wheat damage intensity at 35 of the 144 power stations studied. The stations associated with the largest crop losses differ from those associated with the highest mortality. Co-optimizing for crop gains and mortality reduction slightly increases and meaningfully changes the distribution of social benefits from reducing emissions, highlighting the importance of considering crop losses alongside health impacts when regulating coal electricity emissions in India.

## Significance

India’s wheat and rice crops are critical for its food security but are exposed to high air pollution levels, shown in previous studies to reduce yields. Coal-fired electricity generation is a major contributor to this pollution, but its impact on crop productivity has not previously been quantified. Here, we quantify rice and wheat yield losses associated with coal power stations’ nitrogen dioxide emissions using detailed generation and wind direction data, and satellite measures of nitrogen dioxide and crop productivity. In many regions, yield losses exceed 10%. Per unit of electricity generated, the value of lost crop exceeds monetized pollution-related mortality costs at around half the stations studied. Improved crop productivity is an important cobenefit of reducing coal pollution in India.

India must simultaneously increase food and electricity production to meet its population’s needs, facilitate economic growth, and adapt to climate change (1–3). Despite renewable energy capacity in India growing faster than fossil fuel-based capacity, power generation in India continues to be dominated by coal-fired generators and new coal capacity continues to come online (4). Coal-fired electricity generation is a major contributor to air pollution in India (5), which has been shown to negatively impact crop yields there (6, 7). The use of coal generation to meet growing electricity demand may consequently trade-off against the goal of increasing agricultural productivity to meet rising food demand, but the magnitude of its effect remains unknown. Given India’s role in international grain markets, a quantification of this trade-off is also relevant for global food security (8, 9).

The negative impacts of air pollution on crop productivity have been quantified across a variety of pollutants, crops, and regions. The negative effect of ozone is particularly well-documented (10–20). Researchers have estimated soybean and maize relative yield losses of approximately 5% and 10%, respectively, from ambient ozone exposure in the United States between 1980 and 2011 (12). Early estimates of global yield losses from ozone exposure were estimated between 8.5 to 14% for soybean and 2.2 to 5.5% for maize in 2000, and forecast to rise to 15 to 19% for soybean and 4.4 to 8.7% for maize in 2030 (13, 14). Rice and wheat yields are also similarly impacted: Global relative yield losses were estimated at between 7 to 12% for wheat and 3 to 4% for rice (15), with higher losses for both crops in China (17–19) and for wheat in India (20). The negative effects of other pollutants, including sulfur dioxide (11), nitrogen dioxide (21), and particulate matter (6, 7, 22–25) have also been quantified.

Evidence of yield loss from crop exposure to air pollution at subnational (10, 17, 22, 23), national (6, 11, 19), continental (21, 25), and global scales (13–16) has motivated research on the contribution of emissions from specific sectors to overall losses (26, 27), with particular attention on emissions from energy production (28, 29) and transportation (30, 31).

Studies that assess yield loss from exposure to ambient concentrations of specific pollutants typically use one of two approaches. In the first, chemical transport models (CTMs) are used to estimate spatially explicit concentrations of the pollutants of interest during the growing season, which are combined with previously established exposure–response (ER) relationships (32) to estimate exposure-related yield losses. This approach can enable assessments in regions where ground monitor pollution measurements are unavailable (16–20), large-scale global assessments (13, 15, 24), and analyses of future yield losses under different emissions pathways (14–16), but it must contend with uncertainties in emissions inventories, the CTMs, and the ER functions (15, 33).

The second approach involves regression analysis of long-term records of crop yields, pollution concentrations, and climate variables. This approach is particularly useful when ER functions are unavailable or highly uncertain (6, 7, 10–12, 22), but is limited to regions, pollutants, and crops with sufficiently detailed long-term records. Recent studies have exploited new, high-resolution satellite data to expand the applicability of this approach to questions where the lack of ground monitor data and administrative yield records previously constrained its use (21, 25).

Studies that aim to quantify the impact of specific sources use variations of the two approaches described above. In the first, baseline concentrations are established by a CTM (or a reduced-complexity model) simulation of a comprehensive emissions inventory over the region of interest, and a subsequent simulation that excludes emissions from the sources of interest provides counterfactual concentrations (26, 27, 31). The difference in concentrations can be combined with ER functions to quantify the impact of those specific sources. For the regression-based approach, attributing yield losses to specific sources requires plausibly exogenous changes in their emissions. Power station closures and fuel-switches (e.g.from coal to natural gas) are used as such exogenous shocks (29). Another statistical approach exploits random year-to-year fluctuations in wind direction to generate plausibly exogenous variation in the exposure of receptors to pollution sources. Fluctuations in wind direction are frequently used as the instrumental variable in pollution impact studies that use a 2-stage least squares design (34, 35).

To quantify the impact of coal electricity-related air pollution on crop yields in India, the lack of comprehensive emissions inventories at high spatial and temporal resolution (36, 37), including for electricity generation (38), make a CTM-based approach challenging. A regression-based approach involving closures also cannot be used because while numerous coal-fired units have been retired in the past decade, these retirements have typically been individual electricity generating units at multiunit power stations, and have frequently been accompanied by the commissioning of new units at those power stations, thereby obfuscating any plausibly exogenous signal (39).

The widespread availability of high-resolution wind direction (40) and pollutant concentration data (41) enables us to use a wind variation-based approach to answer this question. Year-to-year fluctuation in wind direction at coal power stations during the growing season creates plausibly random variation in the exposure of cropland receptors to their emissions. This variation can be used to estimate coal-attributable pollutant concentrations at each receptor and, when combined with exposure–response relationships for satellite-measured exposure (21), provide estimates of source-specific yield loss.

In this study, we quantify crop losses from NO_2_ emissions from coal electricity generation in India. We develop a regression model that uses daily generation and wind direction at each power station, satellite measures of NO_2_ concentrations over cropland, and associations between satellite-measured NO_2_ and crop greenness. We estimate our model and the resulting damages for the rice crop grown predominantly in the monsoon, or kharif season, and the wheat crop grown predominantly in the winter, or rabi, season.

We focus on NO_2_ because recent research suggests that it results in high overall yield losses in India (21) through well-documented pathways. Satellite-measured NO_2_ is a good proxy for total NO_*x*_ (42), which also includes nitrogen oxide (NO), and NO_*x*_ is known to affect crop productivity through multiple direct and indirect pathways. NO_*x*_ can be directly phytotoxic by inducing stress from resisting cellular acidification and interfering with the activities of critical enzymes (43, 44). Indirectly, NO_*x*_ is a precursor to ozone formation (45), which itself can directly (46–48) and indirectly (49) reduce crop productivity. NO_*x*_ is also a precursor for particulate matter formation (50, 51), which can reduce crop productivity when the negative impact of dimming exceeds any benefits from increased diffuse irradiance (52, 53). We focus on coal electricity generation because the sector accounts for 30 to 40% of total anthropogenic NO_*x*_ emissions in the country (5, 54, 55) (*SI Appendix*, Fig. S17). An assessment of the impacts of coal NO_2_ emissions may, therefore, illustrate an important lever for improving national and global food security through reducing emissions that have successfully been mitigated in many regions (28, 56).

Although our study focuses on NO_2_-linked yield loss from coal emissions in India, this approach can be extended to other pollution sources with known locations, satellite-measured pollutants, and outcomes. For instance, the same TROPOspheric Monitoring Instrument (TROPOMI) NO_2_ dataset (41) and the region and season-specific concentration–yield relationships (21) can be used to determine power sector-specific yield losses in any geography where we have information on power plant locations and generation.

## Results

### Regression Models to Estimate Coal-Attributable NO_2_ Concentrations.

**Fig. 1.**
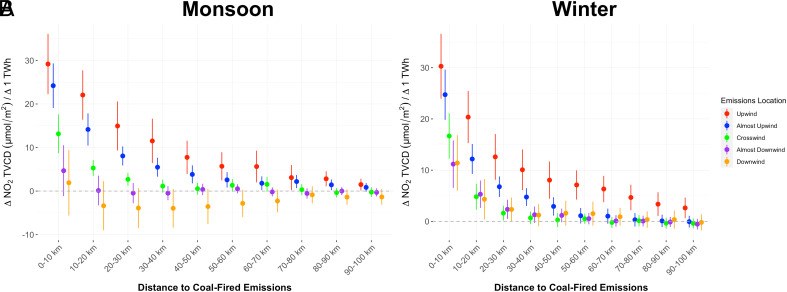
Change in mean seasonal NO_2_ concentrations, measured as the tropospheric vertical column density (TVCD), in the monsoon (*A*) and in the winter (*B*) attributable to changes in wind direction-specific exposure to coal-fired generation at different distances. NO_2_ concentrations over cropland are influenced by changes in exposure to emissions from coal power plants located up to 100 km away. Increased exposure to coal power plants directly upwind has a higher impact than increased exposure in any other direction. The error bars correspond to the 95% CIs for the coefficients using SEs clustered at the state–season level.

We estimate the fraction of observed NO_2_ concentrations from all sources that are attributable to emissions from coal-fired power stations using our regression model. We find that in both the monsoon and winter crop seasons, coal generation emissions significantly affect mean seasonal NO_2_ concentrations above cropland up to 100 km away (Fig. 1).

As shown in Fig. 1, the effect sharply attenuates with distance. In both seasons and for each distance category, the effect of exposure to additional coal generation directly upwind of the cropland is higher than the effect of additional exposure in any of the other directions (see *SI Appendix*, Tables S1 and S2 for detailed regression results).

These estimates are driven by the correlation between the point-level demeaned variation in upwind, “almost” upwind, crosswind, “almost” downwind, and downwind generation, and the point-level demeaned variation in the seasonal NO_2_ mean concentration (*SI Appendix*, Fig. S1). By using extremely local fluctuations in exposure to the emission source and observed NO_2_ concentrations, we minimize the risk of confounding from other sources whose emissions may be correlated with both the generation at the power station and NO_2_ concentrations observed above any given point (see *SI Appendix*, Figs. S2 and S3 for details on the fixed-effects approach).

Exposure to downwind generation in winter has a significant effect on concentrations within 30 km, but the magnitude ranges from around a third to a fifth of that of exposure to upwind generation. In the monsoon, the point-estimates of the effect of additional downwind exposure are negative for certain distances. This should not be interpreted to mean that increasing downwind generation reduces NO_2_ concentrations because the exposure variable is a combination of generation and wind direction. Additional days in a season where the wind blows from a cropland point toward a coal power station will result in greater downwind exposure and are correlated with fewer days of upwind exposure because the total number of days in a season are fixed. The magnitude of these point-estimates is small relative to the upwind point-estimates and none of the negative coefficients are statistically significant at the 5% level.

### State-Level Variation in Coal-Attributable NO_2_ Concentrations.

**Fig. 2.**
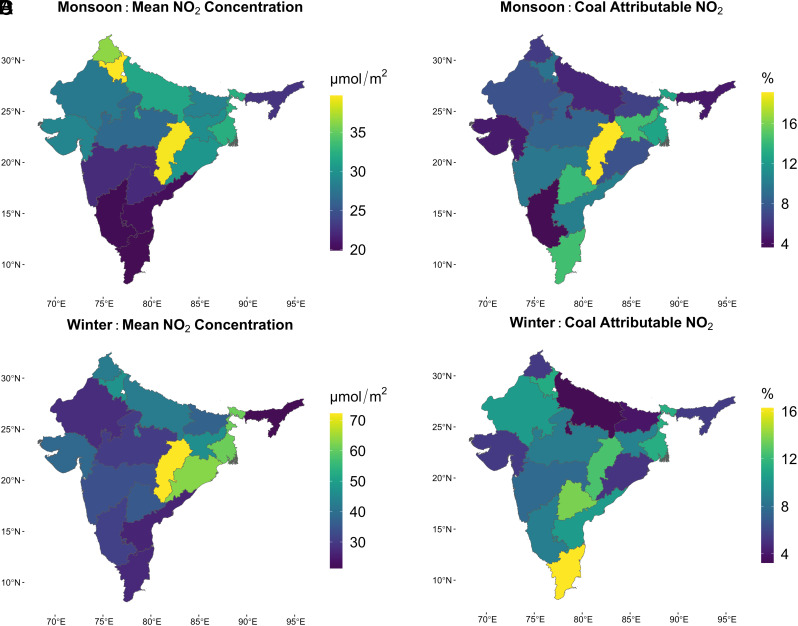
State-level mean seasonal NO_2_ concentrations and the percentage attributable to coal emissions during the 2019 monsoon (Panels *A* and *B*) and winter (Panels *C* and *D*) seasons. Both mean seasonal NO_2_ and the fraction attributable to coal emissions display considerable state-level variation in both seasons. Several states with low mean seasonal concentrations have a high fraction attributable to emissions from coal electricity generation.

Both observed seasonal NO_2_ concentrations and the fraction attributable to coal electricity emissions display considerable state-level variation. Fig. 2 shows state-level seasonal mean NO_2_ concentrations in the monsoon (A) and the winter (C). We find that concentrations vary by more than 2× across states during both seasons. In September–October, mean NO_2_ concentrations are as low as 20 μmol/m^2^ over Tamil Nadu, Karnataka, and Andhra Pradesh, while they approach 40 μmol/m^2^ over Chhattisgarh and Haryana (Fig. 2*A*). Concentrations are uniformly higher across the country in the January–February period that we study for the *rabi* wheat crop. States in the south continue to have relatively low concentrations around 27 μmol/m^2^, while the densely populated Indo-Gangetic plains see high concentrations above 40 μmol/m^2^. West Bengal, Odisha, and Chhattisgarh register averages above 60 μmol/m^2^ (Fig. 2*C*).

In Panels *B* and *D* of Fig. 2, we show the fraction of observed seasonal mean concentrations attributable to coal-fired electricity using our NO_2_ attribution model. We estimate the coal-attributable NO_2_ at each point as the sum of NO_2_ attributable to exposure to coal generation in all directions and at all distances through 100 km and aggregate the results at the state level. Given the combination of coefficients for each direction and the multiple models for different distances, 95% prediction intervals for coal-attributable NO_2_ in each state are estimated using a cluster bootstrap (see *SI Appendix*, Tables S5 and S6 for detailed results).

Across the country, the fraction of observed concentrations attributable to coal emissions varies by around 5×, and this fraction is not always correlated with total seasonal concentrations. There are several states with high seasonal mean NO_2_, where coal emissions do not appear to drive concentrations. In Uttar Pradesh, which is among the top three producers of both kharif rice and rabi wheat (57), NO_2_ concentrations are very high: around 42 μmol/m^2^ in January–February, and approximately 32 μmol/m^2^ in September–October. In both seasons, just around 3.24% (2.15 to 4.01%) and 5.26% (3.84 to 6.35%) of the concentrations, respectively, were attributable to emissions from coal electricity. In such states, it is important to identify specific areas (if any) where controlling coal emissions would meaningfully reduce concentrations and improve crop productivity, as well as conduct future research on quantifying the impact of other NO_*x*_-emitting sectors. In contrast, although Tamil Nadu saw among the lowest mean NO_2_ concentrations, more than 14% was attributable to coal generation in both seasons. In some states with high levels of coal-fired electricity generation, large fractions of high overall NO_2_ concentrations are attributable to coal emissions. In Chhattisgarh, for example, 19.12% (13.96 to 23.04%) and 12.54% (7.74 to 15.25%) of observed concentrations in the monsoon and winter, respectively, are attributable to coal emissions.

State-level averages of coal-attributable NO_2_ concentrations over cropland are consistently, and often substantially, lower than the electricity sector’s ∼30% share in India’s total NO_*x*_ emissions. This finding is consistent with the rapid attenuation in coefficients shown in Fig. 1, and the steep gradients in observed NO_2_ concentrations with increasing distance from thermal power stations noted in previous studies (11, 58). The NO_2_ emitted by a power station rapidly reacts in the atmosphere to form secondary pollutants, and only small amounts of it remain observable as NO_2_ over cropland located further away from the station (58). This suggests that other point and nonpoint sources of NO_2_ emissions located in or closer to cropland may contribute more to NO_2_ concentrations over cropland than their share in total national NO_*x*_ emissions, but quantifying their role is beyond the scope of this study.

### Estimated Yield Gains from Eliminating Coal NO_2_ Emissions.

**Fig. 3.**
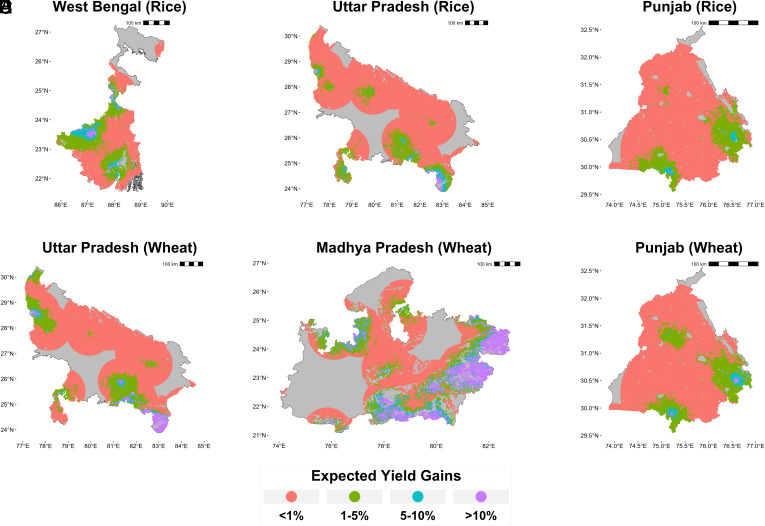
Expected yield gains from eliminating coal-attributable nitrogen dioxide concentrations in major rice (*A*–*C*) and wheat (*D*–*F*) producing states. Large tracts of cropland in all key states are expected to see yield improvements of >1% from eliminating coal-related NO_2_. Data from 2019 growing seasons.

Eliminating NO_2_ emissions from coal would have important effects on yields, albeit the magnitude varies substantially within states. Fig. 3 illustrates this within-state variability in expected yield improvements. Panels *A*–*C* classify each point containing cropland according to the yield change expected from eliminating coal-attributable NO_2_ during kharif season for the three largest rice-producing states (57). Panels *D*–*F* depict the same but during rabi season for the three largest wheat producers (57).

Yield improvements of >10% are expected for the rice crop in western West Bengal (Panel *A*) and south-eastern Uttar Pradesh (Panel *B*) and for the wheat crop in south-eastern Uttar Pradesh (Panel *D*) and eastern Madhya Pradesh (Panel *E*). Yield gains of >5% are expected in several areas near coal power stations in all states. We contextualize these gains by comparing them to long-term yield growth and volatility. Using yields reported during the 10 y period from 2011 to 2020 (59, 60), annual average growth in India’s kharif rice yield was approximately 1.7% per year, and rabi wheat yields grew by approximately 1.5% per year (*SI Appendix*, Fig. S6). The 10% expected gains from eliminating coal NO_2_ emissions in selected regions therefore amount to roughly 6 y of average annual yield growth in both crops. In the same records (59, 60), rice yields fluctuated by a maximum of 5% year-on-year while the maximum change in wheat yields was an approximately 12% drop in 2014 (*SI Appendix*, Fig. S7). The 10%+ expected improvement in wheat yields in selected regions is therefore comparable to the steepest year-on-year drop in yields seen in recent years.

As a fraction of all cropland within 100 km of coal-fired power stations, the largest gains from eliminating coal-attributable NO_2_ are expected for the rice crop in West Bengal and the wheat crop in Madhya Pradesh. Around 5.73% (4.04 to 6.95%) of the cropland within 100 km of coal-fired power stations in West Bengal (Panel *A*) is expected to see yield improvements of between 5 to 10% during the kharif season and 1.66% (1.03 to 2.33%) can expect gains of ≥10%. This is driven by the presence of numerous coal-fired power stations in the Damodar Valley, both in West Bengal and in the neighboring, coal-rich state of Jharkhand. In Madhya Pradesh, the presence of numerous large coal-fired power stations along and across its eastern border with Chhattisgarh drive expected gains of between 5 to 10% on 5.98% (4.66 to 6.42%) of cropland, and gains ≥10% on 11.98% (9.32 to 13.76%) of cropland in the rabi season. The ranges in parentheses correspond to 95% prediction intervals estimated using a cluster bootstrap (see *SI Appendix*, Table S7 for detailed results).

**Fig. 4.**
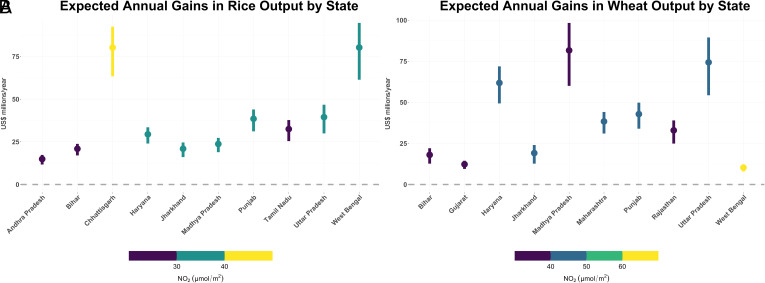
Expected annual gains in rice (*A*) and wheat (*B*) output from eliminating coal emissions across states. The highest absolute gains for rice output occur in West Bengal, and for wheat output in Madhya Pradesh. Data from 2019 growing seasons and wholesale prices of US$600/ton for rice and US$350 for wheat. Error bars indicate 95% prediction interval for monetized state-level output gains, estimated using a state–season cluster bootstrap.

By combining aggregated estimates of yield improvements, reported state-level output (59), and indicative prices to monetize changes in output (61), we find meaningful potential gains from eliminating coal-attributable NO_2_ in several states for both kharif rice and rabi wheat, as shown in Fig. 4.

We monetize potential gains in rice output using an estimated price of US$600/ton (61) (Fig. 4*A*). Potential gains in rice output across all states with some cropland within 100 km of coal power stations are on the order of US$420 million/y. We find that the highest potential gains in rice production using 2019 data are in West Bengal [$80.25 million (61.38 to 94.65)] and Chhattisgarh [$80.2 million (63.38 to 92.47)]. The gains in Chhattisgarh are far greater as a fraction of total output than in West Bengal since it produced on the order of 6.5 million tons of kharif rice in 2019 compared to West Bengal’s 11.3 million tons (59). Potential gains in the other rice-producing states, such as Uttar Pradesh and Punjab, are less than US$50 million/y each.

We monetize wheat gains using an estimated price of US$350/ton (61). For wheat, we see concentrated gains in Madhya Pradesh, Uttar Pradesh, and Haryana. Cumulative increase in wheat output across all states is valued at approximately US$400 million/y.

Since wholesale prices vary across geographies, crop subtypes, and time, we also illustrate state-level output changes that remove the additional uncertainty inherent in monetized estimates (*SI Appendix*, Fig. S12). When aggregated across the ten top states for each crop highlighted in Fig. 4, output gains in percentage terms from eliminating coal emissions are approximately 0.8% (0.65 to 0.98) for kharif rice and 1.1% (0.81 to 1.28) for rabi wheat.

We note that the largest gains in wheat and rice output are not necessarily expected in states with the highest prevailing NO_2_ concentrations, but in locations where coal emissions drive a large fraction of that concentration. Gains are also driven by the amount of crop production in the status quo. Even though coal drives a similarly large fraction of seasonal mean NO_2_ concentrations in Tamil Nadu in both seasons, gains are concentrated in the rice crop because the state does not produce wheat (59).

### Comparing Station-Level Crop and Mortality Damages from Coal Emissions.

**Fig. 5.**
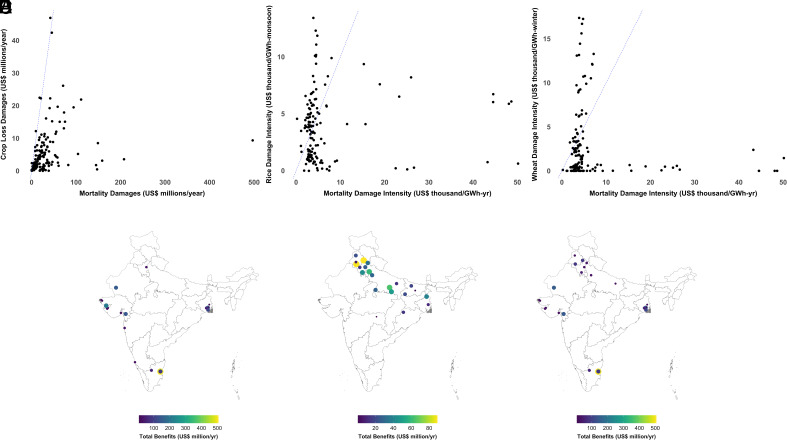
Power station-level variation in absolute crop vs. mortality damages (*A*), rice (*B*), and wheat (*C*) damage intensity vs. mortality damage intensity, and the implications of considering crop damages when identifying optimal emissions reduction policies (*D*–*F*). Panel (*A*) shows that absolute crop damages are considerably lower than mortality damages, but we see in Panels (*B* and *C*) that crop damage intensity during the two growing seasons is higher than mortality damage intensity for several stations. Consequently, different sets of power stations would be prioritized for emissions reductions depending on whether the objective is to minimize mortality (*D*), crop loss (*E*), or a combination of both (*F*). Data for all crop and mortality metrics from 2019.

To assess the magnitude and relative importance of coal’s crop damages, we estimate power station-level crop damages and compare them with the mortality damages associated with each station’s emissions (Fig. 5). We estimate absolute crop damage as the total monetized rice and wheat loss from station-specific generation across both seasons, and the crop damage intensity as the monetized loss per GWh of energy produced during each crop season. We quantify these metrics because they can inform cost–benefit analyses for policy interventions involving the coal fleet. Policies that seek to minimize damages while minimizing the amount of coal generation affected would target stations with the highest damage intensity, while policies that aim to minimize damages while minimizing the number of stations where emissions are cut would focus on stations with the highest absolute damages.

We find large variation in both absolute crop damage and the damage intensity associated with emissions from individual stations. Station-level wheat losses range from $0/y for stations in non-wheat-growing regions to $25.96 (20.97 to 29.97) million per year, and station-level rice losses similarly range from $0/ y to $20.96 (17.53 to 23.46) million per year (*SI Appendix*, Table S8). These estimates use data from 2019, and the ranges in parentheses correspond to the 95% prediction interval estimated using a cluster bootstrap.

Wheat damage intensity goes up to $17,370 (13,280 to 20,480)/GWh of electricity produced during the rabi season, and rice damage intensity goes up to $13,420 (10,590 to 15,520)/GWh of electricity produced during the kharif season (*SI Appendix*, Table S9). As a result of the variation in crop damage intensities, a small number of units drive total losses in each season. Approximately 20% of coal-fired electricity generation in the 2019 kharif season was associated with 50% of total coal NO_2_-linked rice loss, and only 12% of total rabi generation was linked to 50% of total wheat loss.

To compare crop impacts with mortality impacts, we use estimates from previous work on unit-level premature mortality associated with NO_2_, SO_2_, and PM_2.5_ emissions for each of over 500 individual units representing approximately 90% of the Indian coal fleet (62). To facilitate a comparison between crop loss and mortality impacts, we monetize premature mortality using a Value of Statistical Life (VSL) of Rs. 10.3 million (approximately US$120,000) (4).

We find that absolute crop damages are considerably lower than mortality damages (Fig. 5*A*). Absolute crop damages do not exceed US$50 million per year at any coal power station, while several power stations are estimated to result in over US$100 million per year of mortality damages. Almost all stations are located to the right of the line of equality in Fig. 5*A*, indicating that the absolute mortality damages are higher than absolute crop damages.

However, we find that the intensity of crop damage in both seasons is comparable and frequently higher than mortality damage intensity (Fig. 5 *B* and *C*). Rice damage intensity is higher than mortality damage intensity at 58 (41 to 70) power stations and wheat damage intensity is higher than mortality damage intensity at 35 (25 to 53) power stations out of the 144 stations for which both crop and mortality intensity estimates are available. The ranges in parentheses are computed by comparing the lower bound of crop damage intensity to the upper bound of mortality damage intensity, and the upper bound of crop damage intensity to the lower bound of mortality damage intensity, respectively, and are not CIs.

For both crops, crop damage intensity exceeds mortality damage intensity by the widest margin for power stations in the northern agricultural states of Haryana and Punjab. The mortality intensity of these stations is on the order of 0.035 deaths/GWh, which equates to a damage intensity of approximately US$4,200/GWh. In comparison, the rice damage intensity associated with their emissions is approximately 3× more, on the order of US$13,000/GWh and the wheat damage intensity is around 4× more, on the order of US$17,000/GWh (*SI Appendix*, Table S9). These results are necessarily sensitive to the prices of rice and wheat and the VSL, so we also illustrate station-level differences in terms of the percentile occupied by each station along the two dimensions (*SI Appendix*, Figs. S13 and S14).

The divergence in trends between absolute damages and damage intensity is because of the difference in the generation that is linked to crop and mortality impacts. Crop damages are linked to seasonal generation (January–February for the rabi wheat losses and September–October for the kharif rice losses), while mortality damages are associated with annual generation and its modeled impact on annual average PM_2.5_ concentrations. The crop damage intensities shown in Panels *B* and *C* of Fig. 5 therefore apply only to generation in the respective seasons, while the mortality damage intensity applies to generation throughout the year.

The inclusion of crop damage externalities can meaningfully affect the distributional consequences of policies aimed at minimizing pollution-related damages from electricity generation. To illustrate this, we identify power stations where annual emissions should be eliminated (e.g. through early retirement) to minimize mortality damages (Fig. 5*D*), crop damages (Fig. 5*E*), and the combination of mortality and crop damages (Fig. 5*F*), subject to only targeting 10% of total annual generation. Our results show that the choice of objective strongly influences which regions will incur the costs and enjoy the benefits of reduced emissions. When the objective is solely to minimize mortality, power stations predominantly in Gujarat, West Bengal, Tamil Nadu, and Rajasthan are selected. If the sole objective is to minimize crop loss, power stations in Punjab, Haryana, Uttar Pradesh, Bihar, and West Bengal are selected. In the scenario where we optimize for combined mortality and crop damages, the highest priority stations include high crop damage intensity stations in Punjab and Haryana along with the most mortality intensive stations in West Bengal, Tamil Nadu, and Gujarat.

Explicitly accounting for crop damages also has the potential for slightly increasing the total social benefits expected from eliminating emissions from a fixed fraction of total generation. In the scenario where we only optimize for mortality damages (Fig. 5*D*), total benefits from targeting 10% of generation are US$1.97 billion per year (1.72 to 2.14), including US$1.91 billion/y (1.67 to 2.07) from reduced mortality and US$58.19 million/y (45.26 to 68.06) from increased crop output. In the co-optimization scenario (Fig. 5*F*), we expect total benefits of US$2.02 billion/y (1.75 to 2.2), of which US$1.81 billion (1.59 to 1.97) derive from reduced mortality and US$202.9 million (161.3 to 235) from increased crop output. In the co-optimization scenario, eliminating annual emissions from 10% of annual generation delivers approximately one-quarter of the US$820 million of kharif rice and rabi wheat losses attributable to NO_2_ from coal electricity generation.

We illustrate the impact of accounting for crop damages using a base case of eliminating annual emissions. This is because quantifying the mortality impacts of generation occurring in the four months relevant for crop damages would require subannual air quality models and mortality ER functions and is beyond the scope of this study. To illustrate the potential of highly targeted policies that limit emissions during specific seasons, we assume that the mortality intensity is uniform for generation throughout the year. Under this assumption, we note that absolute crop damages are much closer in magnitude to seasonal absolute mortality damages (*SI Appendix*, Fig. S15). Co-optimizing for mortality and crop damage reduction by only targeting emissions during the four months linked to crop loss in this study, while still constrained to only targeting 10% of annual generation as before, we find similar total benefits of approximately US$2.01 billion. However, crop gains of around US$745 million are a larger share of the total, and season-specific emission controls are found to be optimal all across the fleet (*SI Appendix*, Fig. S16).

## Discussion

Our study of the effects of coal-attributable NO_2_ on crop productivity in India contributes to two active areas of research. First, we build on the extensive literature on the impact of air pollution on crop growth by quantifying the impact of a prominent source of anthropogenic emissions (5) in a region important for global food security (8, 9). Previous studies have extensively examined the impact of different pollutants on crop productivity in India (6, 7, 13–15, 20, 22, 23), but assessments of the role of electricity emissions have largely been limited to other geographies (11, 28, 29).

Since our approach captures multiple pathways of NO_2_’s impact on crop productivity, our estimates of the crop impacts of coal NO_2_ emissions must be compared with previous estimates of the all-source impacts of multiple pollutants. Our estimates can most directly be compared to estimates of the impacts of satellite-measured NO_2_ concentrations on wheat yields in India (21). In that study, the authors estimated that halving winter NO_2_ concentrations could increase wheat yields by 6.4% (SE = 1.2%). Here, using data from 2019 we find that eliminating NO_2_ emissions from coal generation during the winter can increase output by approximately 1.1% (0.81 to 1.28) across the 10 largest wheat-producing states that accounted for 98% of rabi wheat production in 2019 (59).

Electricity generation accounts for between 30 to 40% of anthropogenic NO_*x*_ emissions (5) but closer to 20% of total NO_*x*_ emissions that also include biogenic emissions (55), so if coal NO_*x*_ emissions were associated with wheat damage proportional to their share of total NO_*x*_ emissions, they would result in around a 2.5% loss. Winter NO_*x*_ emissions from coal generation therefore account for slightly less than half the wheat yield loss that their contribution to total NO_*x*_ emissions would suggest. The discrepancy between coal electricity’s share of NO_*x*_ emissions and its share of total estimated NO_2_-linked yield loss is explained by the difference between the sector’s share of emissions and its share of yield-relevant NO_2_ concentrations over cropland discussed previously in the context of Fig. 2. The difference may partly be explained by the sharp attenuation in coal-attributable NO_2_ concentrations with increasing distance from coal power stations (Fig. 1). As a result of that drop, NO_2_ concentrations over large tracts of winter cropland appear minimally influenced by coal NO_2_ emissions. NO_2_ emissions from coal may still indirectly impact yields in these areas through their role in forming secondary pollutants (58, 63), but the NO_2_ coefficient we use cannot explicitly account for these effects.

Given that NO_*x*_ is an ozone (45) and aerosol precursor (50, 51), we also place our coal-specific estimates in the context of the total impact of ozone and aerosols on crop yields in India. We caution that these effects are not easily compared because the coefficient that we use to estimate the yield effects of coal-attributable NO_*x*_ does not fully capture its indirect effects on yield through its complex role in ozone and aerosol formation (21, 45, 50, 51).

Estimates of total ozone-linked wheat losses in India are highly uncertain and range between 5 and 40% (20), which suggest that coal NO_*x*_ emissions could explain up to a fifth of ozone-linked losses. Previous estimates of the combined effects of ozone and aerosols on wheat yields in India are in the region of 30% (7, 64). The yield losses estimated here are around 3% of those combined effects.

Recent studies estimate ozone-related rice yield losses in India of between 7.18 to 12.42 million tons per year between 2005 and 2020 (65). Here, we estimate coal-linked losses of 0.63 Mt (0.5 to 0.74) across the ten largest states for rice output, which is between 5% and 9% of total losses (65). The combined effects of ozone and aerosols on kharif rice were estimated at −18% in an earlier study but were not statistically significant (7). Similar to wheat, the coal and NO_*x*_-specific effect estimated in this study is a small fraction (∼5%) of the total, but we note again that our NO_*x*_ coefficient does not fully capture its ozone and aerosol-mediated impacts on rice productivity.

Our study also adds to the literature on the externalities of coal-fired electricity generation in India (66, 67). Prior studies have primarily focused on estimating the impact on human health through estimates of increased premature mortality (4, 62, 68), and all-encompassing climate damages that are frequently monetized using a social cost of carbon (69, 70). Our results show that damages in the form of lost crop output, while lower than monetized estimates of mortality damages in the aggregate, are highly heterogeneous across regions (Fig. 3) and power stations (Fig. 5). Integrated cost–benefit assessments for interventions (66) such as mandating pollution controls or early retirements of coal-fired generators should explicitly include crop yield impacts since they can constitute a meaningful share of the social costs of coal emissions. This may be particularly important since recent research has found that mortality costs alone are unlikely to bridge the gap between the operational costs of coal-fired power stations in India and cleaner alternatives (71).

We note four important limitations in our analysis that present opportunities for further research. First, although our attribution model addresses many sources of confounding, it structurally cannot exclude the effects of other noncoal NO_2_ emission sources in the same direction as a given coal-fired power station. We account for the challenge posed by multiple colocated coal-fired power stations by only including cropland that has a single coal-fired power station at a given distance and check that our findings are robust to the removal of power stations colocated with the largest sources of potentially confounding NO_*x*_ emissions (*SI Appendix*, Fig. S5), but our coefficients remain susceptible to including the impact of colocated noncoal emissions. Future research may seek to combine TROPOMI NO_2_ observations (42) and geocoded NO_*x*_ emissions inventories (5) to directly quantify the effect of noncoal sources.

Second, the results are subject to the same limitations as prior findings that we use to translate attributable NO_2_ to changes in crop productivity (21). The approach used to estimate the association between NO_2_ exposure and crop productivity captures the net impact of numerous processes that are challenging to directly observe. The total effect likely includes the direct phytotoxicity of NO_2_ (72), of the O_3_ formed in High VOC:NOx regimes (73, 74), and the radiation-altering effects of nitrate aerosols (75, 76). The combined effect is sufficient for estimating cumulative damages from coal electricity emissions, but future studies may be able to separate different mechanisms using satellite measures that can be globally scaled.

Third, the crop damages estimated here are likely underestimates since they do not consider other air pollutants, and the effects of coal generation through non-air-pollution channels such as water stress and heavy metal contamination (77–79). They also do not consider other crops, including the substantial rabi rice crop in states like West Bengal, Andhra Pradesh, and Telangana (59).

Finally, comparing coal’s crop damages with more extensively studied mortality damages remains challenging because of differences in pollutant species considered and the time scales over which the damages occur. Premature mortality estimates are typically based on changes in annual average PM_2.5_ (4, 62) while our crop loss estimates are based on changes in NO_2_ concentrations over specific growing seasons. Further research on the human health impacts of seasonal emissions from coal power stations will allow for a better assessment of the tradeoffs and complementarities between increasing crop productivity and improving human health for policies that reduce emissions during specific periods of the year.

Despite the need for additional research, our results identify and quantify one important lever for improving crop yields through improving air quality in a highly polluted region important for global food security, add to our understanding of the spatially heterogeneous externalities of coal-fired electricity generation, and demonstrate an approach that can use satellite measures of pollution to assess the impact of emission reductions on myriad outcomes of interest.

## Materials and Methods

### Integration of Satellite Observations and Electricity Generation Data.

We integrate five main data sources: daily NO_2_ Tropospheric Vertical Column Density (TVCD) observations from the TROPOMI (42), daily electricity generation time series from India’s Central Electricity Authority (80), daily Near-Infrared reflectance of vegetation (NIRv) estimates computed using reflectance bands from Moderate Resolution Imaging Spectroradiometer (81), daily wind directions, temperature, and precipitation from the ERA5-Land Reanalysis dataset (40), and a cropland mask from the European Space Agency (ESA) WorldCover dataset (82).

All satellite-derived quantities are extracted from Google Earth Engine with a pixel resolution of 1 km, which corresponds to the approximate pixel size of the Level 3 tropospheric NO_2_ TVCD band that we use for estimating NO_2_ concentrations. The native resolution of the daily TROPOMI NO_2_ data, made available as a Level 2 product, is approximately 5.5 km × 3.5 km at nadir (42). The higher spatial resolution in the Level 3 product is achieved by time-averaging values across the crop season and regridding to 1 km × 1 km resolution. Previous research has noted better agreement with in situ NO_2_ observations when TROPOMI Level 2 measurements are regridded to 1 km × 1 km (41). The ESA land use mask is available at a 10 m resolution (82), and we include in our dataset all 1 km points where any portion of the point is classified as cropland. We retain the fraction of each point that is classified as cropland to appropriately weight quantities when extrapolating our results. ERA-5 Land Reanalysis values are available at a resolution of approximately 10 km, which results in multiple neighboring 1 km grid cells having similar seasonal precipitation and temperature values (40).

To compute seasonal, direction-specific, exposure to coal-fired generation for each point, we first compute seasonal totals of generation at each coal-fired power station for each of eight wind directions at the power station: north, north-east, east, south-east, south, south-west, west, and north-west. Assuming a stable NO_2_ emission factor for each generating unit, this quantity is proportional to the exposure to that station’s NO_2_ emissions delivered to cropland in a given direction. Season here refers to the September–October period in a given year for monsoon exposure, and the January–February period for winter exposure. As an example, for the 2019 monsoon season, for every coal-fired power station c, we compute eight quantities (denominated in GWh) corresponding to the amount of generation occurring at c on days between September 1, 2019, and October 31, 2019, when the wind direction was d (*SI Appendix*, Fig. S1*B*).

Next, we compute the bearing from each point to all coal-fired power stations in each of the ten distance categories, starting with 0 to 10 km through 90 to 100 km. Total generation at c on days when the wind blew from direction d at the plant is considered upwind generation at point p if the bearing from p to c matches d (so, if the power station is north of the point, generation at the power station on days with northerly winds there is classified as upwind generation).

Total generation in adjacent octants is considered “almost” upwind, generation in their neighboring octants is flagged as crosswind, as “almost” downwind in their neighbors, and finally generation in the opposite octant is classified as downwind generation. For that example point where the power station is to the north, generation on days where the power station saw north-easterlies or north-westerlies is considered “almost” upwind, generation on days with easterlies and westerlies is classified crosswind, on days with south-easterlies and south-westerlies as “almost” downwind, and as downwind on days with southerlies when the wind is expected to carry pollutants away from the point (*SI Appendix*, Fig. S1*B* illustrates the classification of exposure into octants).

### Regression Model for NO_2_ Attribution.

To estimate the impact of coal electricity generation on tropospheric NO_2_ concentrations above cropland, we regress the seasonal mean NO_2_ concentration over each point on the cumulative generation during the season in each of the eight directions. We include controls for temperature and precipitation, and point and season-state fixed effects (FE), where season corresponds to a specific two-month period like September–October 2018, not “monsoons” in general:

[1] $$\begin{array}{c} \begin{array}{cc} \;{\text{NO}}_{2,i,t} & =\beta_{\mathrm{up}}\cdot Gen_{up,i,t}+\beta_{up^{\prime }}\cdot Gen_{up^{\prime },i,t}\\ & \;+\beta_{\mathrm{cross}}\cdot Gen_{cross,i,t}+\beta_{down^{\prime }}\cdot Gen_{down^{\prime },i,t}\\ & \;+\beta_{\mathrm{down}}\cdot Gen_{down,i,t}+\beta_{W}\cdot W_{i,t}\\ & \;+p_{i}+c_{s,t}+\epsilon_{i,t} \end{array}, \end{array}$$

This model is estimated separately for winter and monsoon and for exposures at each distance category to more flexibly capture the shape of the coal-attributable NO_2_ gradient under different weather conditions. For both the monsoon and the winter, we include five years of observations from 2018 through 2022. In each model, we only include points exposed to a single coal power station at that distance to reduce the risk of confounding (see *SI Appendix*, Tables S3 and S4 for details on the number of points excluded).

The point FE controls for time-invariant unobservables that are correlated with both generation exposure and NO_2_ concentrations over a given cropland point. The season-state FE controls for time-varying unobservables that affect generation exposure and NO_2_ concentrations across the state. Consequently, the model only relies on the correlation between deviation from mean generation exposure and the deviation from mean NO_2_ concentration at each point, after also excluding any variation that similarly affects generation exposure and NO_2_ concentrations across the state in that season (see *SI Appendix*, Figs. S2 and S3 for illustrations of the fixed-effects approach).

We note that these coefficients can be biased by nonelectricity NO_*x*_ emissions colocated with coal-fired power stations, such as other heavy industry or transportation activity (5). We assess this risk by quantifying the extent to which noncoal NO_*x*_ emissions are colocated with coal power stations (*SI Appendix*, Fig. S4). We find only a weak relationship between proximity to coal power stations and noncoal anthropogenic NO_*x*_ emissions. Since we do find a negative relationship, we ensure our results are robust to this colocation by re-estimating our models after excluding power stations (and cropland in the 100 km catchment area around them) in the top quartile of colocated noncoal NO_*x*_ emissions (*SI Appendix*, Fig. S5). To ensure that no single region is driving the results, we separately estimate the model for four subregions (*SI Appendix*, Figs. S8 and S9). Owing to much smaller sample sizes when considering individual regions, the effects are more uncertain, and typically not significant all the way through 100 km, but the direction and magnitude of upwind exposure coefficients are similar. We similarly estimate the model for different sizes of power stations to ensure that the results are not being driven only by large emitters (*SI Appendix*, Figs. S10 and S11).

### Estimating Yield Gains from NO_2_ Reduction.

To estimate potential yield gains from reducing NO_2_ emissions from coal power stations, we first estimate the potential reduction in seasonal mean NO_2_ concentrations from eliminating coal-attributable NO_2_. For a given season t and exposure distance d, this is estimated as follows:

[2] $$\begin{array}{c} \begin{array}{cc} Coa\hat{l}NO_{2,i} & ={\hat{\beta }}_{up}\cdot Gen_{up,i,t}+{\hat{\beta }}_{up^{\prime }}\cdot Gen_{up^{\prime },i,t}\\ & \;+{\hat{\beta }}_{cross}\cdot Gen_{cross,i,t}+{\hat{\beta }}_{down^{\prime }}\cdot Gen_{down^{\prime },i,t}\\ & \;+{\hat{\beta }}_{down}\cdot Gen_{down,i,t} \end{array}\;, \end{array}$$

Since points can be exposed to coal generation at different distances, we sum coal-attributable NO_2_ from across all distance categories. Points can also be exposed to multiple coal power stations within each distance category, so the generation terms in the equation above include all generation occurring in a given distance category and not just that from a single power station.

To translate the estimated reduction in seasonal mean NO_2_ concentrations to changes in yield, we use coefficients estimated in an earlier study which examined the association between seasonal TROPOMI NO_2_ concentrations and NIRv (a measure of crop greenness), and estimated region-specific coefficients (21). We use an India-specific $\beta_{\;{\text{NO}}_{2}}$ coefficient of 0.0006 (SE = 0.0001) for monsoon rice and 0.0007 (SE = 0.0002) for winter wheat, which refers to the reduction in NIRv associated with a 1 μmol/m^2^ increase in NO_2_ concentration (21). Estimating the change in yield requires specifying a relationship between NIRv-measured greenness and yield. Using results from prior studies that demonstrate a linear relationship between NIRv and yield (83, 84), and a baseline NIRv of 0.007 that corresponds to zero crop growth (85), we estimate the expected percentage change in yield in season t as follows:

[3] $$\begin{array}{c} \Delta Y\hat{i}eld_{i}=\frac{\;{\text{NIRv}}_{i}+(Coa\hat{l}NO_{2,i}\cdot \beta_{\;{\text{NO}}_{2}})-0.007}{\;{\text{NIRv}}_{i}-0.007}-1. \end{array}$$

### Estimating Output Gains Across States.

To estimate absolute gains in output, of potential relevance to cost–benefit analyses of interventions that reduce electricity-sector emissions, the changes in yield need to be applied to location-specific estimates of current yield or output. We use state-level output estimates from India’s Ministry of Agriculture and Farmers Welfare (59) in combination with our state-level cropland-weighted yield change estimates in order to estimate absolute changes in output from eliminating coal-sourced NO_2_:

[4] $$\begin{array}{c} \begin{array}{cc} & \Delta O\hat{u}tput_{s,t}=\\ & Output_{s,t}\cdot \frac{\sum_{i}Coa\hat{l}NO_{2,i}\cdot \beta_{\;{\text{NO}}_{2}}\cdot CropFrac_{i}}{\sum_{i}\;{\text{NIRv}}_{i}-0.007\cdot CropFrac_{i}}\\ & \;\;\;\;\;\;\;\forall i\in s \end{array}, \end{array}$$

The cropland fraction is estimated with the total cropland in the state in the denominator, and not the total cropland in the state within 100 km of coal-fired power stations. Using the latter would overestimate output gains since the state-level output estimates are from all cropland in the state.

The output gains are monetized using prices of US$600/ton for rice and US$350/ton for wheat (61). These are highly approximate values that do not reflect the variation in grain prices over time and geography, and across different varieties of rice and wheat, and are only intended to indicate the order of magnitude of agricultural output gains from eliminating coal-sourced NO_2_ pollution. We illustrate the nonmonetized estimated gains in tons of rice or wheat in *SI Appendix*, Fig. S12.

### Estimating Damages and Damage Intensity Across Power Stations.

Power station-level damages are estimated similarly to expected state-level damages, except with NO_2_ and yield changes for each point estimated separately for every power station p that the point is exposed to:

[5] $$\begin{array}{c} \begin{array}{cc} & \Delta Da\hat{m}ages_{p,t}=\\ & \sum_{s}Output_{s,t}\cdot \frac{\sum_{i}Coa\hat{l}NO_{2,i,p}\cdot \beta_{\;{\text{NO}}_{2}}\cdot CropFrac_{i}}{\sum_{i}\;{\text{NIRv}}_{i}-0.007\cdot CropFrac_{i}}\\ & \;\;\;\;\;\;\;\;\forall i\in s \end{array}, \end{array}$$

The damages are summed over all states because NO_2_ emissions from a power station may affect crop productivity outside the borders of the state in which the power station is located. We then combine damages estimated separately for both rice and wheat to obtain cumulative crop damages because mortality and other damages that crop damages may be compared with are full-year damages, not season-specific ones (Fig. 5*A*).

Crop-specific damage intensity (Fig. 5 *B* and *C*) is estimated by dividing power station-level damages by the amount of total electricity generation in that season (January–February 2019 for the wheat damage intensity, and September–October 2019 for the rice damage intensity):

[6] $$\begin{array}{c} \Delta Damag\hat{e}\;Intensity_{p,t}=\frac{\Delta Da\hat{m}ages_{p,t}}{Total\;Generation_{p,t}}, \end{array}$$

As before, power station damages and damage intensity are monetized using prices of US$600/ton for rice and US$350/ton for wheat to facilitate comparisons with other, noncrop damages associated with coal-fired electricity generation.

### Estimating Uncertainty in Coal-Attributable NO_2_ Concentrations and Yield Losses.

To estimate the yield loss attributable to coal NO_2_ at a point, we combine estimates of attributable NO_2_ there from coal exposure in eight directions and across up to ten distance categories, and previously estimated coefficients that relate NO_2_ exposure to crop greenness. Given the multiple sources of uncertainty, we use a cluster bootstrap (86) with 2,500 simulations (one set of simulations each for monsoon and winter) to estimate 95% prediction intervals around the quantities of interest.

In each iteration of the bootstrap, we sample state–season (e.g. “Uttar Pradesh–Monsoon 2019”) combinations with replacement from the full dataset. We sample clusters, rather than individual rows, so the size of the dataset on which the ten distance-specific models are fit varies across iterations. After sampling clusters, we filter down to observations from points exposed to a single coal power station (as in the base case) and estimate each of the ten distance-specific models. We use the resulting coefficients to estimate counterfactual NO_2_ concentrations by zeroing out coal exposure in all directions and at all distances for all cropland points in the full, nonsampled, dataset.

We use the point-level counterfactual NO_2_ concentrations to estimate four quantities of interest within the bootstrap. State-level coal-attributable NO_2_ concentrations (Fig. 2 *B* and *D*) are computed as the state-level average difference between observed and counterfactual NO_2_ concentrations in 2019. For yield-related metrics, we compute point-level impacts on rice and wheat yields in the monsoon and winter bootstrap, respectively, using Eq. **3**. To reflect uncertainty in the coefficients relating NO_2_ exposure to greenness, we sample from a Gaussian with the mean and SE values described above (21) instead of using the mean estimates of 0.0006 for rice and 0.0007 for wheat. We aggregate these point-level yield impact estimates to compute state-level cropland fractions expected to see different levels of yield gains from eliminating coal emissions (Fig. 3), state-level output gains (Fig. 4), and station-level damages (Fig. 5).

The bootstrap procedure provides 2,500 estimates of the four quantities. For example, we generate 2,500 estimates of coal-attributable NO_2_ concentrations for each of the states shown in Fig. 2, and of the fraction of cropland in each yield gain category for each crop and state shown in Fig. 3. For each quantity, we use the 2.5th and 97.5th percentile values from the n= 2,500 distribution to estimate the 95% prediction interval.

### Comparing Crop and Mortality Damages and Identifying Optimal Emissions Reductions.

Comparing station-specific crop and mortality damages is challenging because of differences in the pollutant species considered as well as the differing time periods over which they are estimated. Prior studies that estimate the mortality damages from coal emissions typically do so using estimates of annual changes in pollutant concentrations and the resulting relative risk of premature mortality (4, 62). Here, we estimate crop damages associated with emissions that occur during two, two-month windows.

We address the challenge of differing time periods when considering combined damages in the base case (Fig. 5*F*) by computing annual average crop damage intensity over the full year *y* (here *y*= 2019) rather than just the crop season *t* as in Eq. **6**:

[7] $$\begin{array}{c} \Delta Damag\hat{e}\;Intensity_{p,y}=\frac{\Delta Da\hat{m}ages_{"p,y}}{Total\;Generation_{p,y}}, \end{array}$$

This likely understates crop damage intensity by not considering emissions impacts on summer crops (21) and the rabi rice cultivation that occurs in several regions (59).

To identify power stations where NO_2_ emissions should be eliminated to maximize only mortality reduction benefits while limiting affected generation to less than 10% of total annual generation (Fig. 5*D*), we rank power stations for which both mortality and crop damage intensity estimates are available in descending order of mortality intensity (62) and select stations until they cumulatively account for 10% of total annual generation in 2019. Total social benefits are estimated by combining mortality benefits monetized using a Value of Statistical Life estimate of US$120,000 (4) and monetized estimates of crop gains using 2019 data and reference prices of US$600/ton for rice and US$350/ton for wheat (61). To optimize solely for crop productivity gains (Fig. 5*E*), we rank order power stations in descending order of annual average crop damage intensity estimated in Eq. **7** and repeat the remaining steps. For the co-optimization scenario in Fig. 5*F*, we sum monetized mortality damage intensity and annual average crop damage intensity and rank power stations in descending order of combined annual damage intensity before selecting the most damaging 10% of generation.

We also illustrate the potential for seasonal emissions reductions (*SI Appendix*, Fig. S16). To compute optimal season-specific emissions reductions, we consider each combination of power station and season a separate emissions reduction opportunity. We compute total damage intensity as the sum of mortality damage intensity and the specific crop damage intensity relevant for that season. In the absence of season-specific mortality damage intensity estimates, we assume they are the same in both seasons. These mitigation opportunities are again ordered in descending order of combined damage intensity and all opportunities are selected until their cumulative generation accounts for 10% of total annual generation. We select up to 10% of total annual generation instead of 10% of generation in the monsoon and winter seasons to illustrate an alternate approach to attaining similar total social benefits to those attained from all-year interventions in the base case (Fig. 5*D*–*F*).

To quantify uncertainty in combined mortality and crop yield benefits from reduced emissions, we construct a range. The lower end is the sum of station-level 2.5th percentile values of rice and wheat output damages from our study and the lower estimate of mortality damages from previous research (4, 62). The upper end is the sum of station-level 97.5th percentile values of monetized rice and wheat damages and the higher estimate of mortality damages. This range should not be interpreted as a prediction interval.

## Supplementary Material

Appendix 01 (PDF)

## Data Availability

R code and datasets have been deposited in Zenodo (DOI: 10.5281/zenodo.13958688) (87).
