# Supplementary material for: Quantifying the impact of air pollution from coal-fired electricity generation on crop productivity in India
Source: Proc Natl Acad Sci U S A. 2025 Feb 3;122(6):e2421679122. doi: 10.1073/pnas.2421679122 (PMC11831135; doi:10.1073/pnas.2421679122)
Supplement: Supplementary file 1 — Appendix 01 (PDF) [file pnas.2421679122.sapp.pdf]

Supporting Information for

Quantifying the impact of air pollution from coal-fired electricity generation on crop productivity in India

Kirat Singh, David B. Lobell and Inês M.L. Azevedo

Kirat Singh.
E-mail: ks676@stanford.edu

This PDF file includes:

Figs. S1-S17
Tables S1-S9.

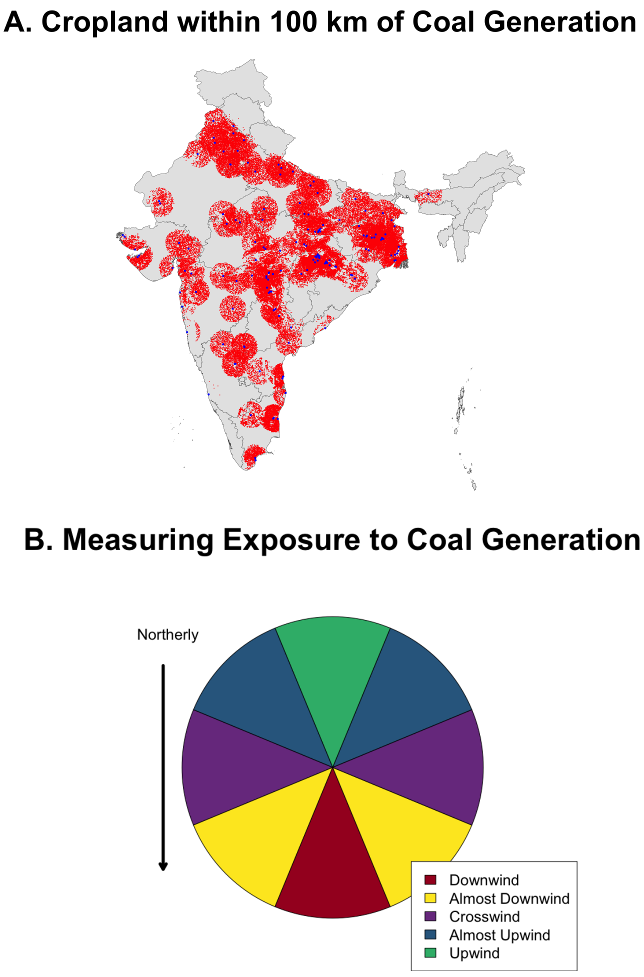

Fig. S1. Cropland within 100 km of operational coal-fired power stations in India (Panel A), and an illustration of the wind direction-based exposure measurement approach (Panel B). Although coal-fired power stations (shown in blue) are concentrated near coal mines in eastern India, large tracts of cropland (in red) across the country lie within 100 km of these stations. To compute exposure to coal generation in a season, we consider daily generation and wind direction at each coal-fired power station. If the wind direction at a station is northerly (Panel B), cropland in the octant directly south of the station will be exposed to that day's generation worth of Upwind exposure. Cropland in adjacent octants will be exposed to the equivalent amount of "Almost Upwind" exposure, and so on.

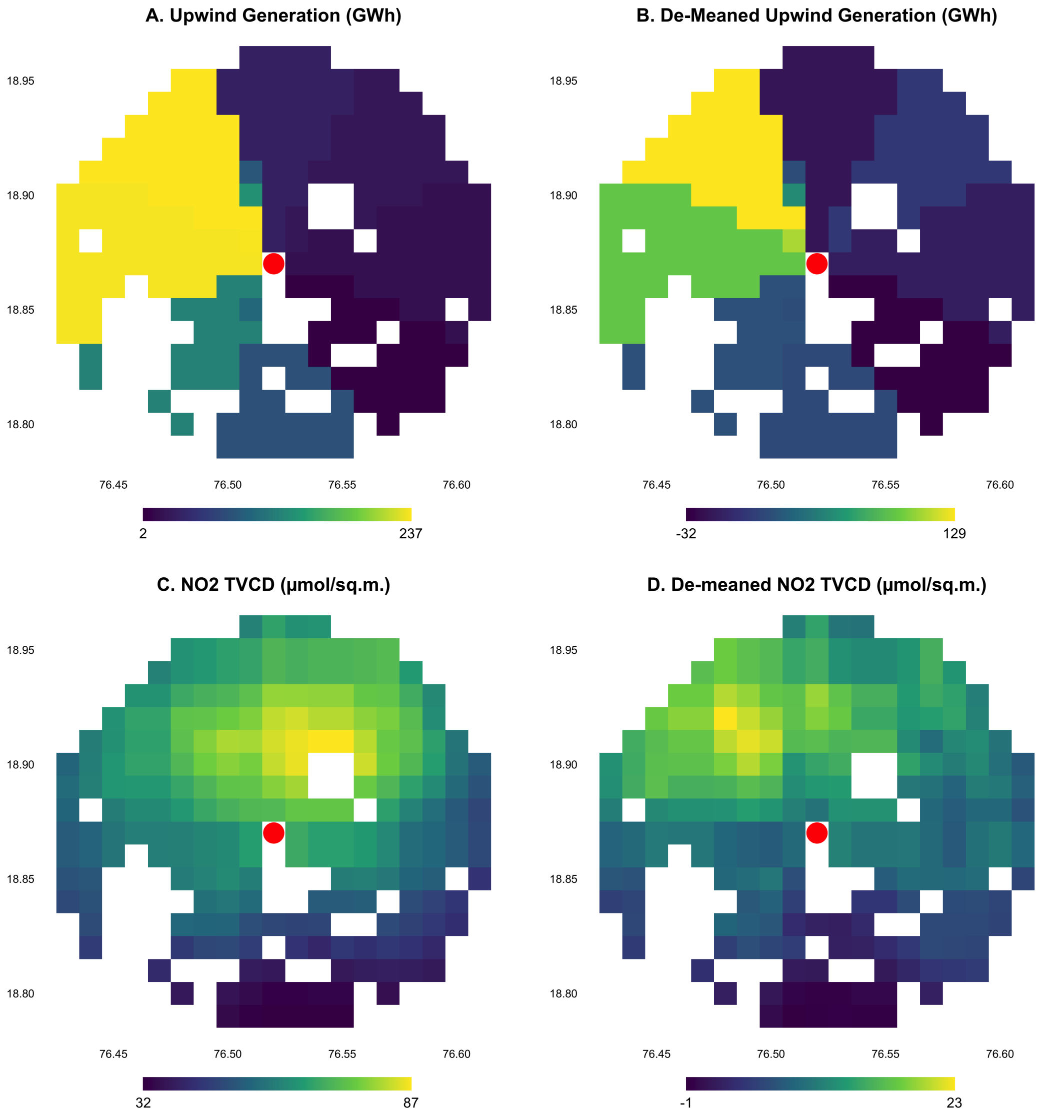

Fig. S2. Illustration of the fixed-effects approach for a single power station, distance and exposure direction. Panel A shows the mean exposure to upwind generation during the winter season for cropland within a 10 km radius of the Parli Thermal Power Station (shown in red at the center) in Maharashtra using data from 2018-2022. On average, cropland to the west and north-west of the Station has the highest exposure to upwind generation (and emissions). In the January-February 2021 (Panel B), cropland to the north-west has atypically high exposure. Similarly, Panel C shows mean winter season NO_2 concentrations, which are typically highest to the north-east of the Station centroid. In 2021 (Panel D), the concentrations in the north-west are the highest relative to the mean concentrations in Panel C. The regression model uses the time-demeaned quantities shown in Panels B and D to estimate the association between seasonal exposure and concentration to minimize the impact of confounding variables that exert an influence on the mean concentrations shown in Panels A and C.

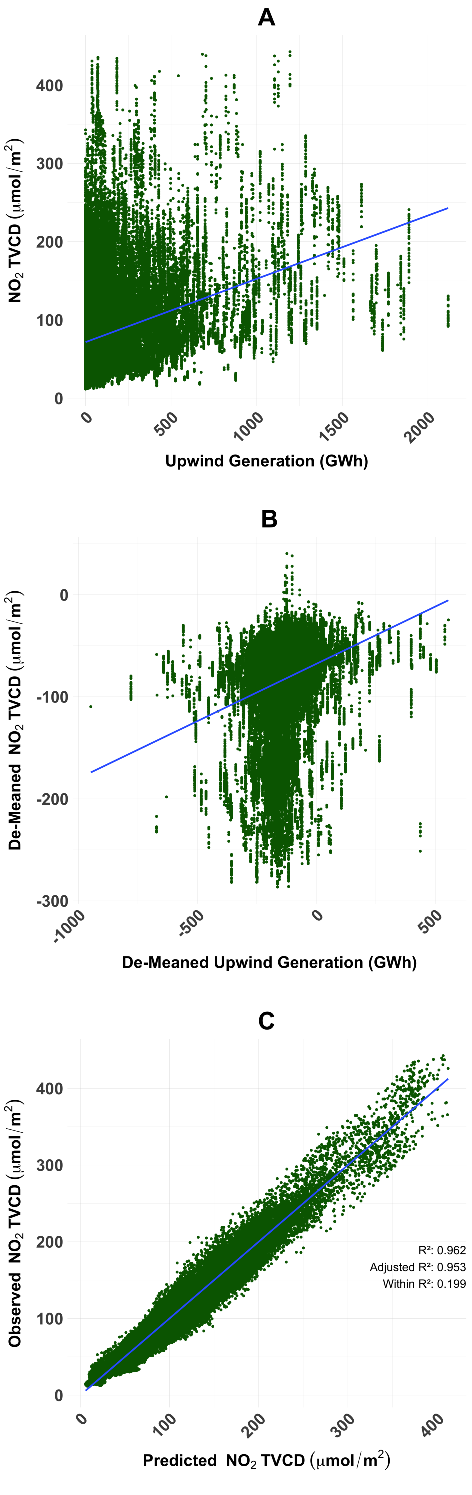

Fig. S3. Illustration of the fixed-effects approach using the 10 km model. Panel A shows a scatterplot of mean winter NO_2 concentrations for all cropland points exposed to a single coal power station within 10 km. Each cropland point appears 5 times in this plot - once for each winter season between 2019 and 2023. In Panel B, both values are de-meaned to remove point-level means across the five years (the point-level fixed-effect) and state-season means in a given year (the state-season fixed-effect). The de-meaned NO_2 concentrations now represent at each point the deviation from the multi-year average concentration at that point and excludes any changes in NO_2 concentrations that occur state-wide in a given winter. De-meaned exposure to upwind generation similarly represents, for each point, the deviation from typical upwind generation exposure at that point with any changes in exposure that occur state-wide. These de-meaned quantities (along with the other exposure and meteorological covariates that are analogously de-meaned) are used in the regression model. Panel C shows the relationship between observed mean winter NO_2 concentrations and concentrations predicted by the 10 km regression model. The R^2 of 0.962 corresponds to the R^2 of the simple linear fit shown in blue in Panel C. The within-R^2 of 0.19 refers to the ability of the model to explain variation in the outcome variable after removing fixed-effects.

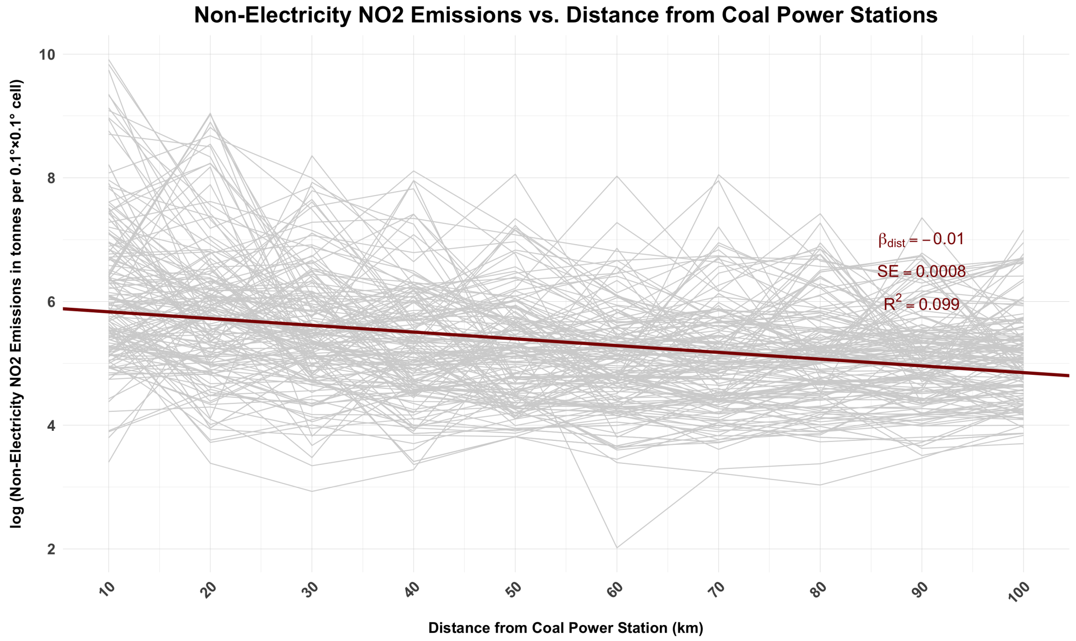

Fig. S4. Relationship between distance from known coal power stations and non-electricity NO_x emissions. Using annual estimates of anthropogenic NO_x emissions from EDGAR at the 0.1 $\deg \times$ 0.1 $\deg$ level, we estimate average, non-electricity sector, anthropogenic NO_x emissions per 0.1 $\deg \times$ 0.1 $\deg$ in each 10 km annular circle through 100 km for each of 168 coal power stations. We regress log(average emissions) on distance to estimate the percentage change in non-electricity NO_x as we move further from coal power stations. Moving 1 km out is associated with an approximately 1% reduction in non-electricity NO_x emissions (red trend-line). Grey lines show trends for each individual power station.

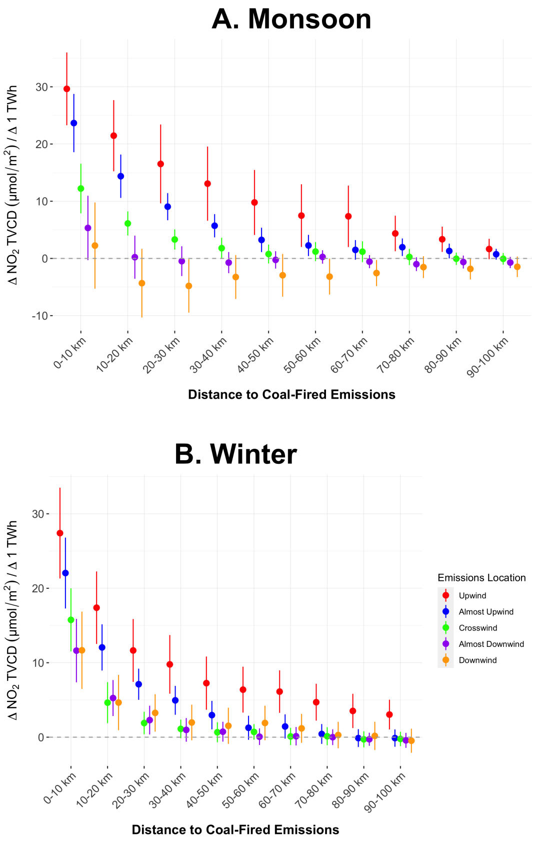

Fig. S5. Effects of coal generation at multiple distances for the monsoon and winter crop, estimated after excluding the top quartile of power stations in terms of non-electricity NO_x emissions in a 10 km radius. We find similar coefficients to the models estimated using the full dataset but with higher uncertainty on account of a smaller number of observations, suggesting that the results are not driven by other NO_x emissions in the vicinity of coal power stations.

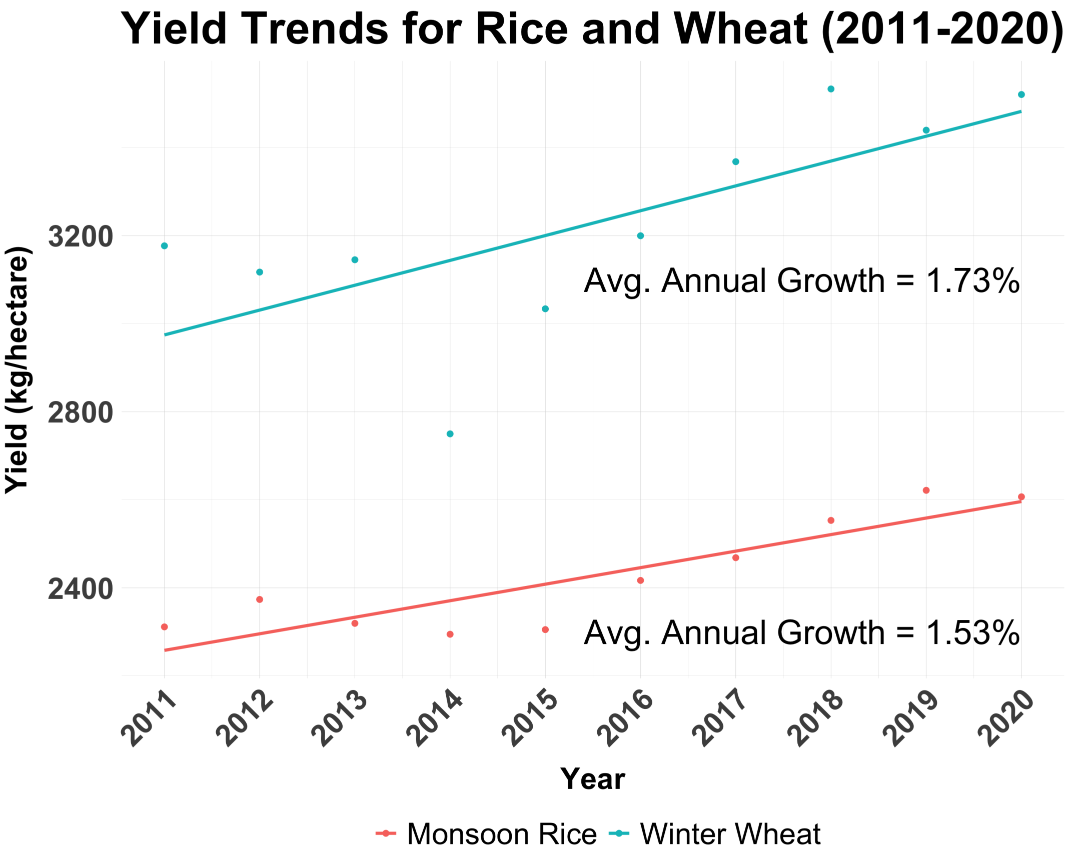

Fig. S6. Trends in India's rice and wheat yields between 2011-2020. Average yields for both crops have grown during this period. Wheat yields have increased slightly faster (1.73% per year on average) than rice yields (1.53% per year on average).

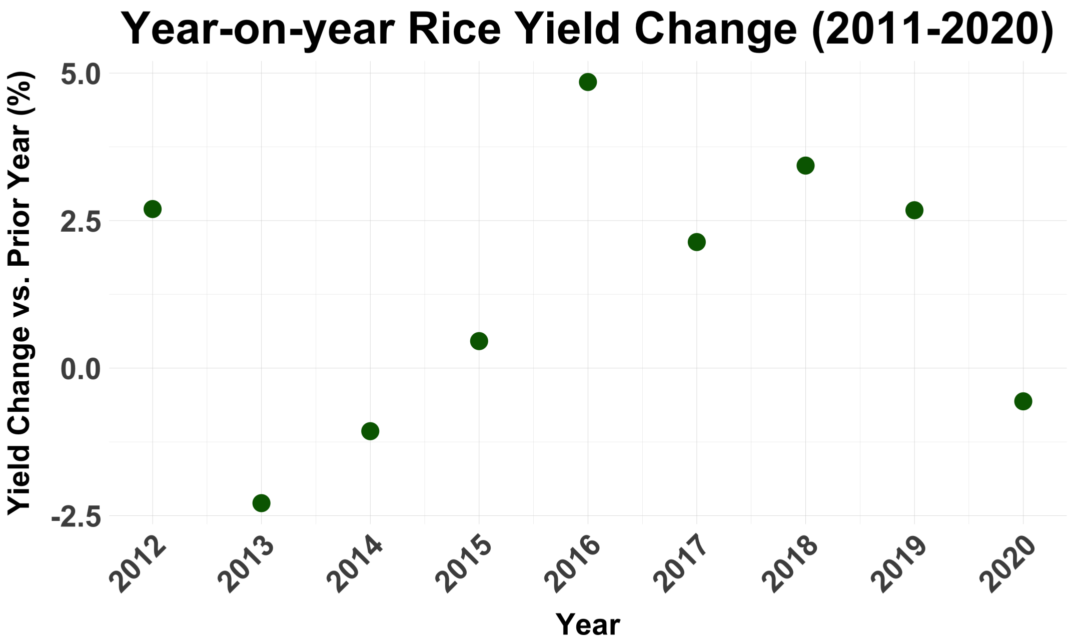

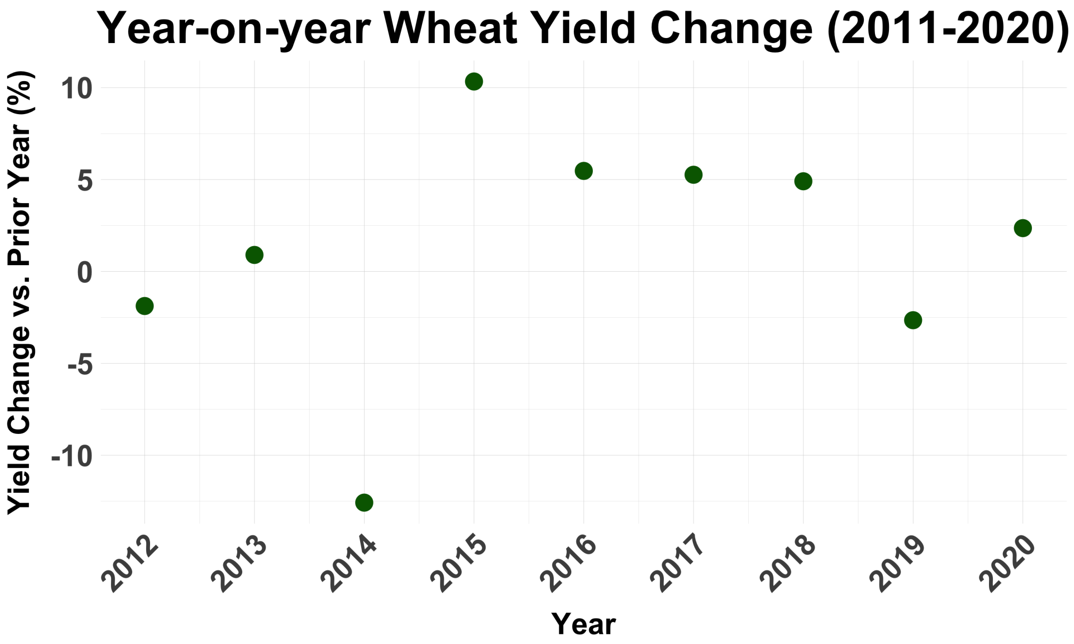

Fig. S7. Year-on-year changes in India's *kharif* rice and *rabi* wheat yields between 2011 and 2020. Rice yields have fluctuated less than wheat yields - while the maximum change in rice yield was +5% between 2015-2016, wheat yields dropped by around 12% in 2014 before recovering by around 11% the subsequent year.

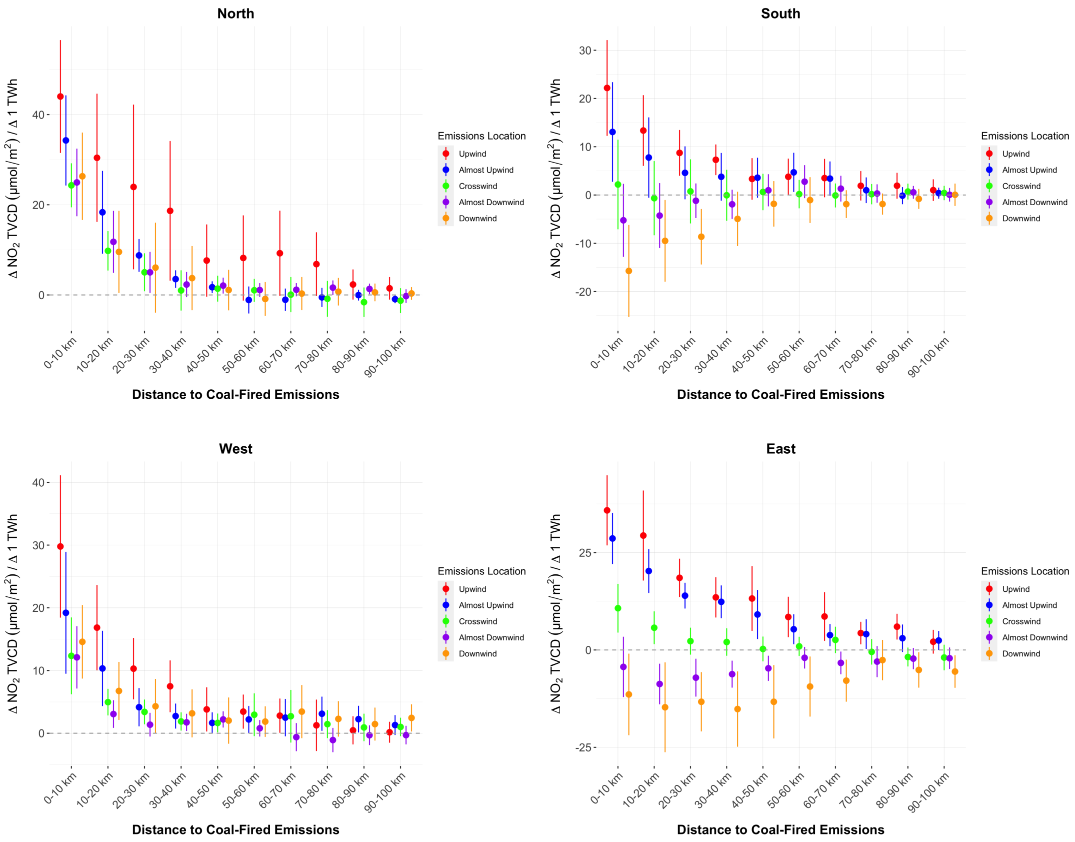

Fig. S8. Effects of coal generation at multiple distances for the monsoon crop, estimated separately for each region. The size of the estimated effects are broadly similar across regions, with slightly larger sizes estimated in the north, and similar to the effects seen in the base case regression that combines observations from all regions. The region-specific estimates are more uncertain.

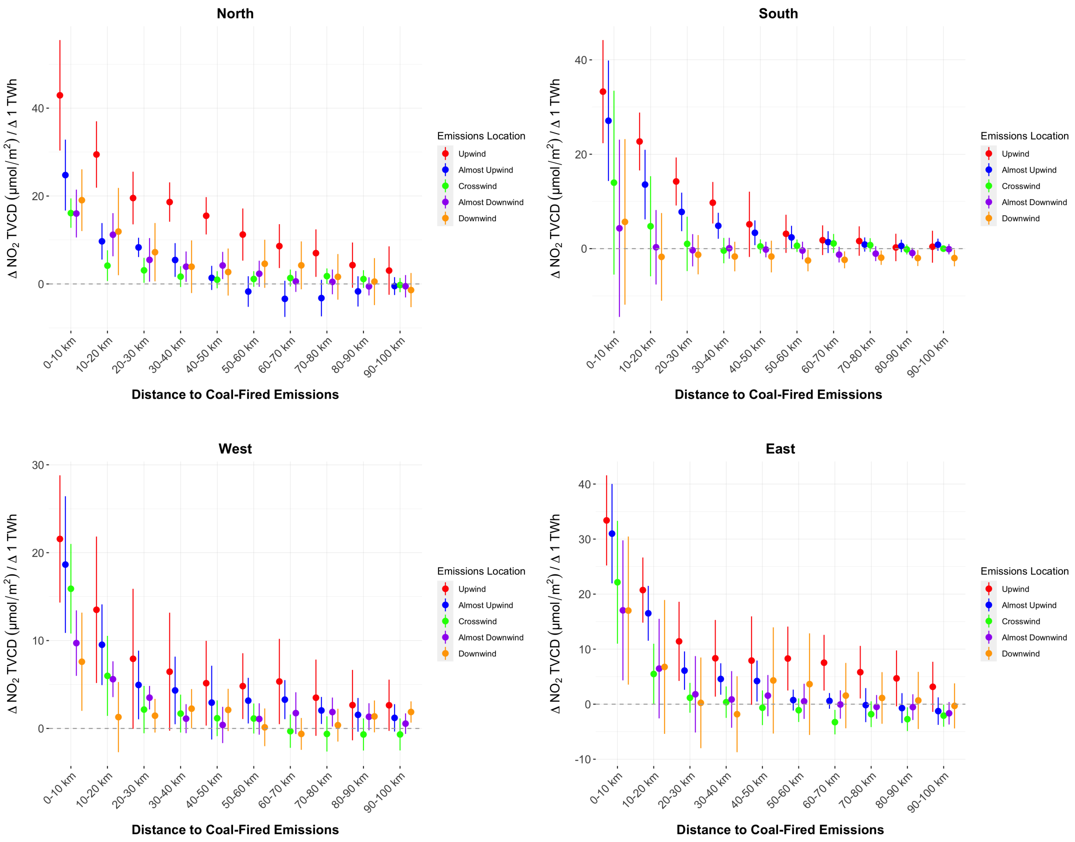

Fig. S9. Effects of coal generation at multiple distances for the winter crop, estimated separately for each region. The effects are similar in magnitude to the base case, upwind exposure has a consistently larger impact than exposure from any other direction, but the larger standard errors mean that the effects of upwind generation are not significant in some regions all the way through 100 km.

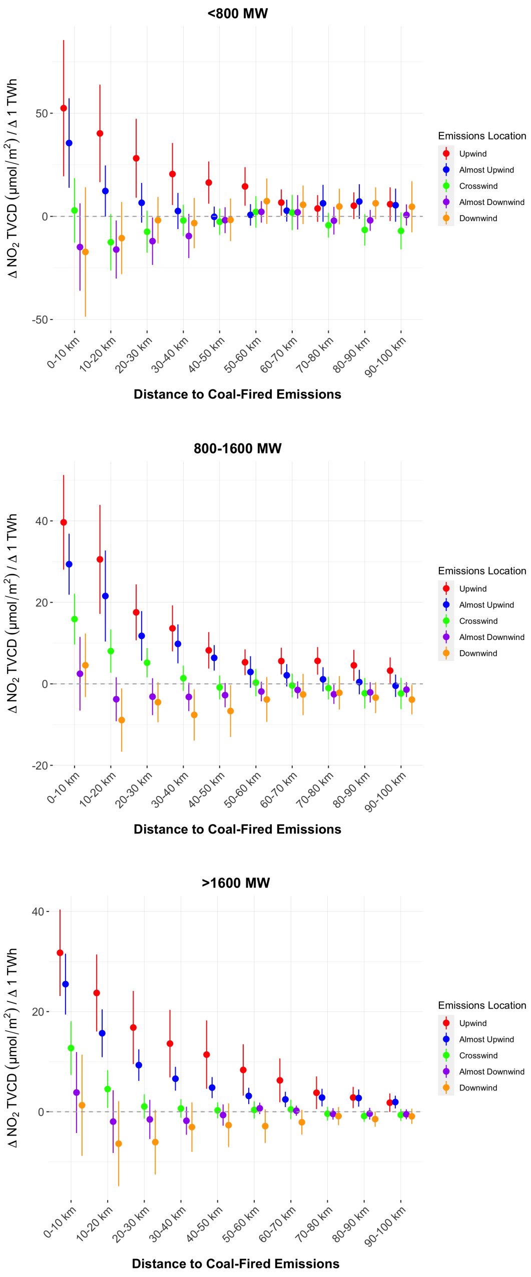

Fig. S10. Effects of coal generation at multiple distances for the monsoon crop, estimated separately for different sizes (in terms of installed capacity, in MW) of coal power stations. The capacity cut-offs are selected to approximately divide the 189 power stations into three equally-sized groups. There are 69 stations in the 'small' category, 57 in the 'medium' category, and 63 in the 'large' category.

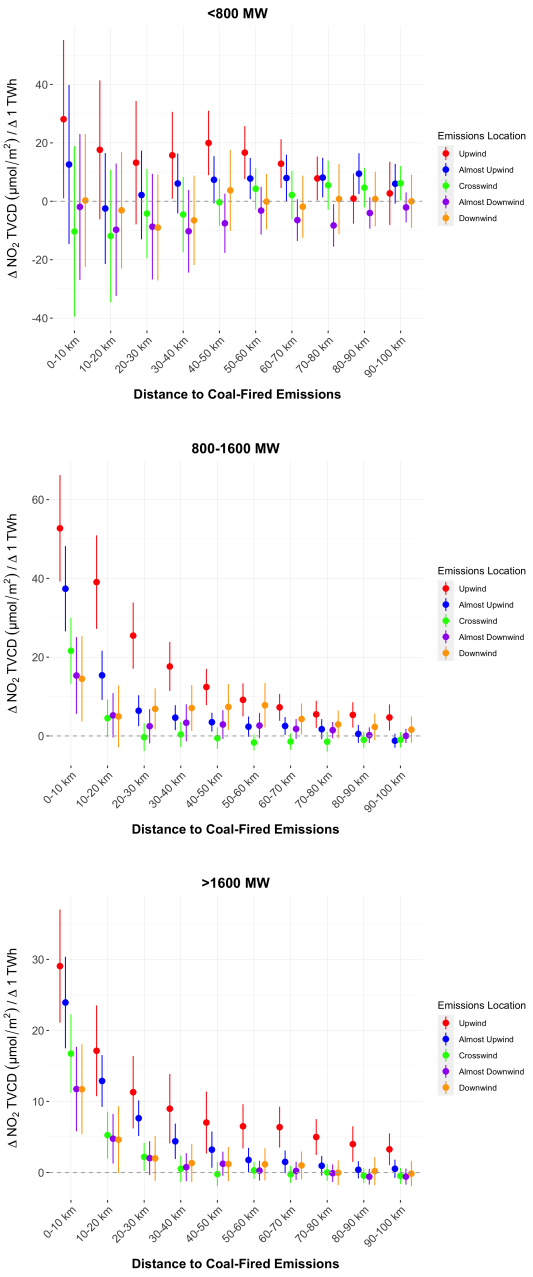

Fig. S11. Effects of coal generation at multiple distances for the winter crop, estimated separately for different sizes (in terms of installed capacity, in MW) of coal power stations. The capacity cut-offs are selected to approximately divide the 189 power stations into three equally-sized groups. There are 69 stations in the 'small' category, 57 in the 'medium' category, and 63 in the 'large' category.

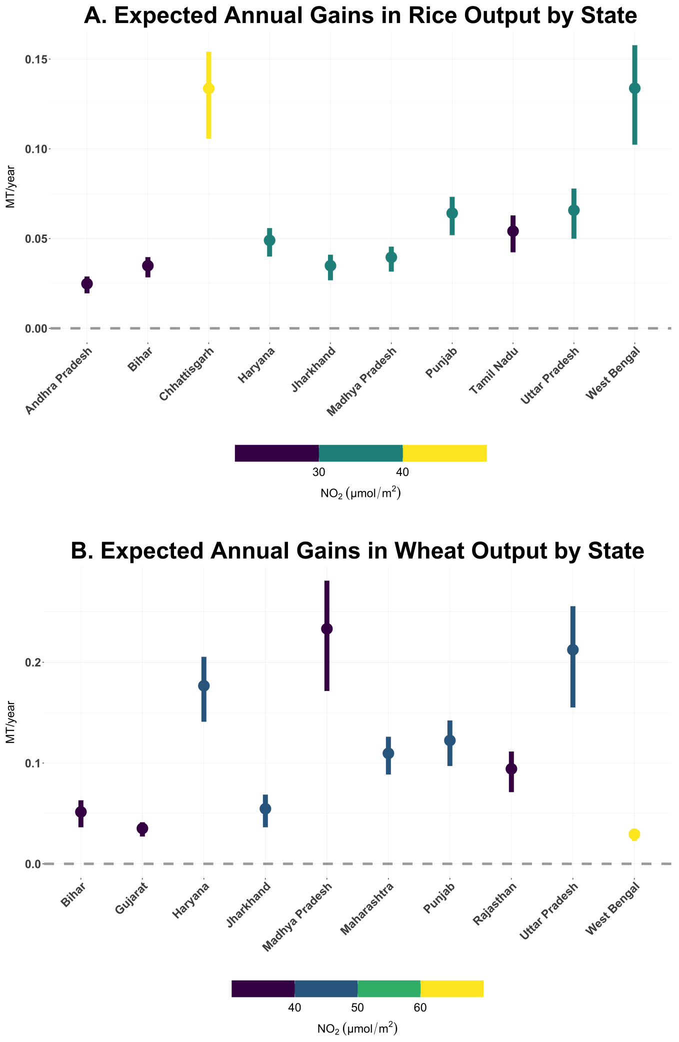

Fig. S12. Expected gains in rice and wheat output from eliminating coal-related NO_2 emissions, expressed in million tonnes/year. We illustrate state-level gains in terms of output to address the additional uncertainty introduced by the use of wholesale market prices to monetize pollution damages.

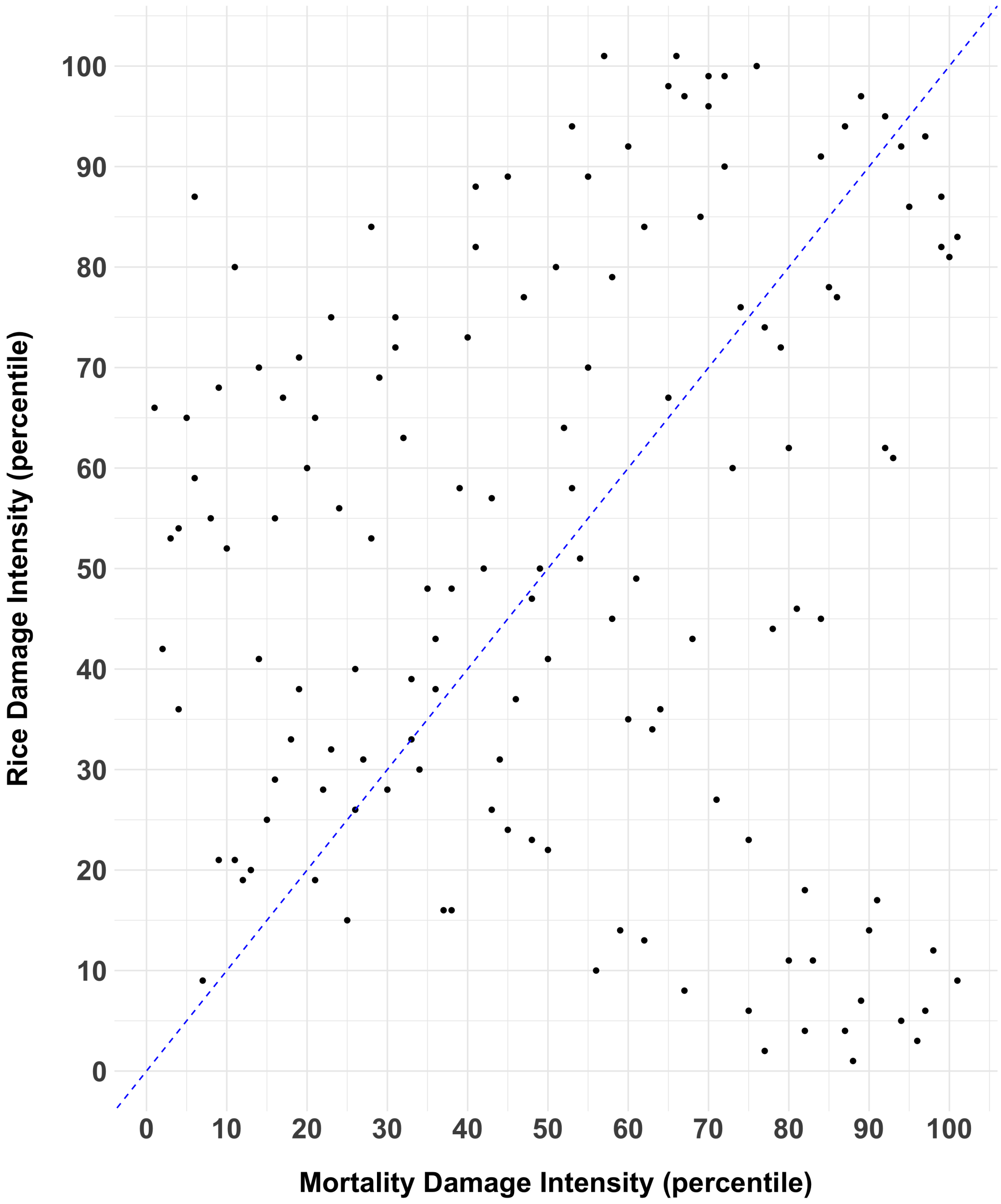

Fig. S13. Percentile of rice damage intensity vs. percentile of mortality damage intensity for individual power stations. Power stations above the 45-degree rank higher in terms of crop damage intensity compared to where they rank in terms of mortality damage intensity. Both quantities are estimated using data from 2019.

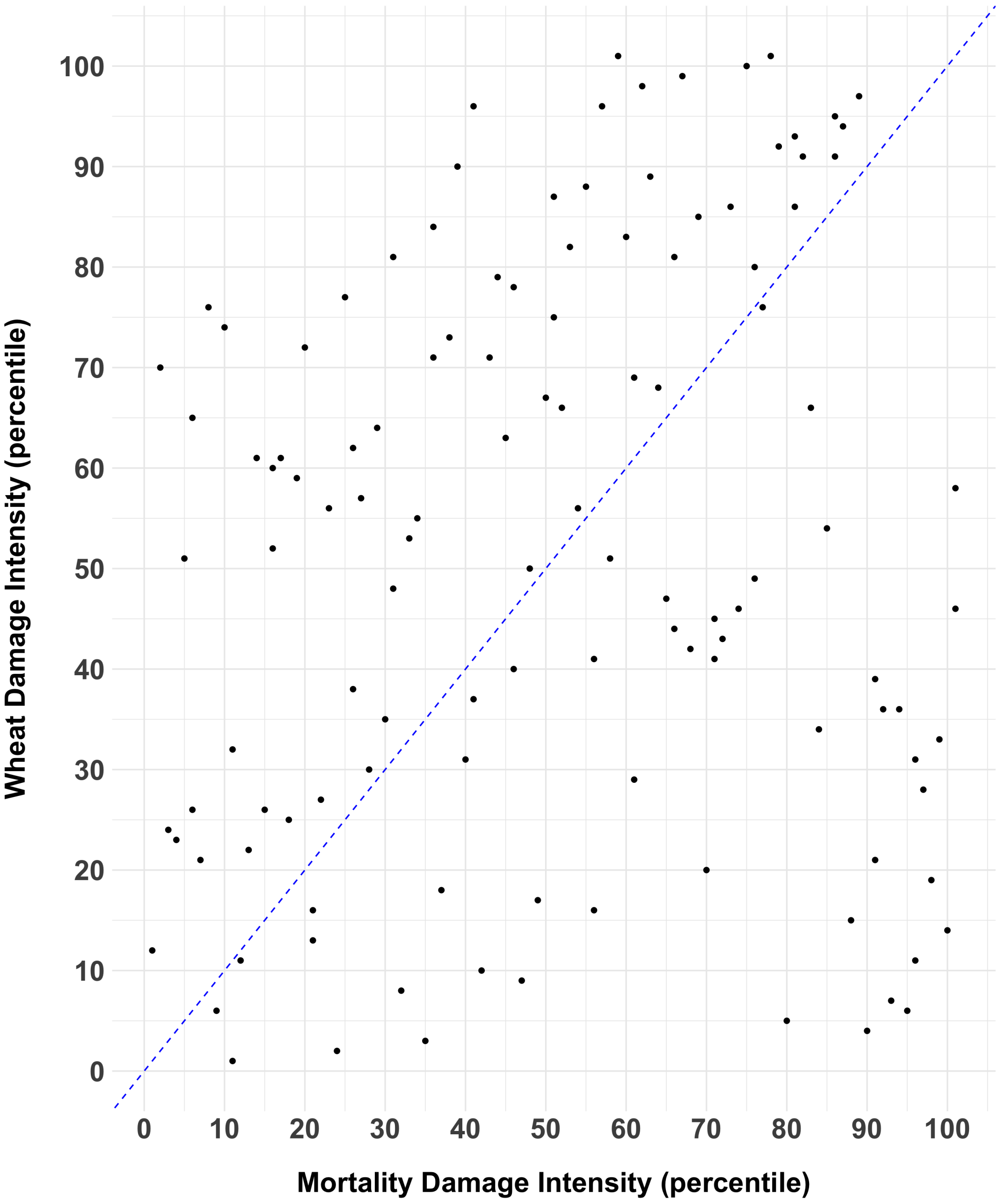

Fig. S14. Percentile of wheat damage intensity vs. percentile of mortality damage intensity for individual power stations. Power stations above the 45-degree rank higher in terms of crop damage intensity compared to where they rank in terms of mortality damage intensity. Both quantities are estimated using data from 2019.

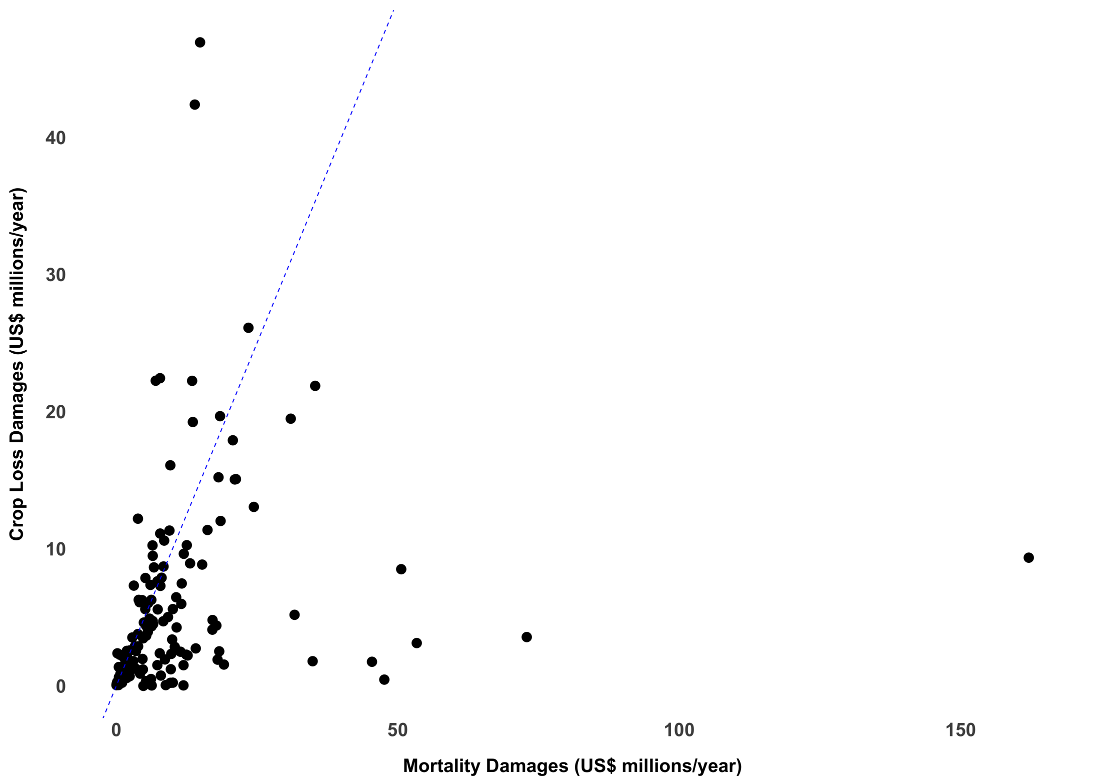

Fig. S15. Absolute crop damages vs. absolute mortality damages only considering mortality damages from crop season-specific generation. By applying the mortality intensity of generation to only the generation occurring in the period where it is found to result in NO_2-related crop damages, we can compare station-specific crop and mortality damage reduction from eliminating emissions during crop seasons. Both quantities are estimated using data from 2019.

Kirat Singh, David B. Lobell and Inês M.L. Azevedo

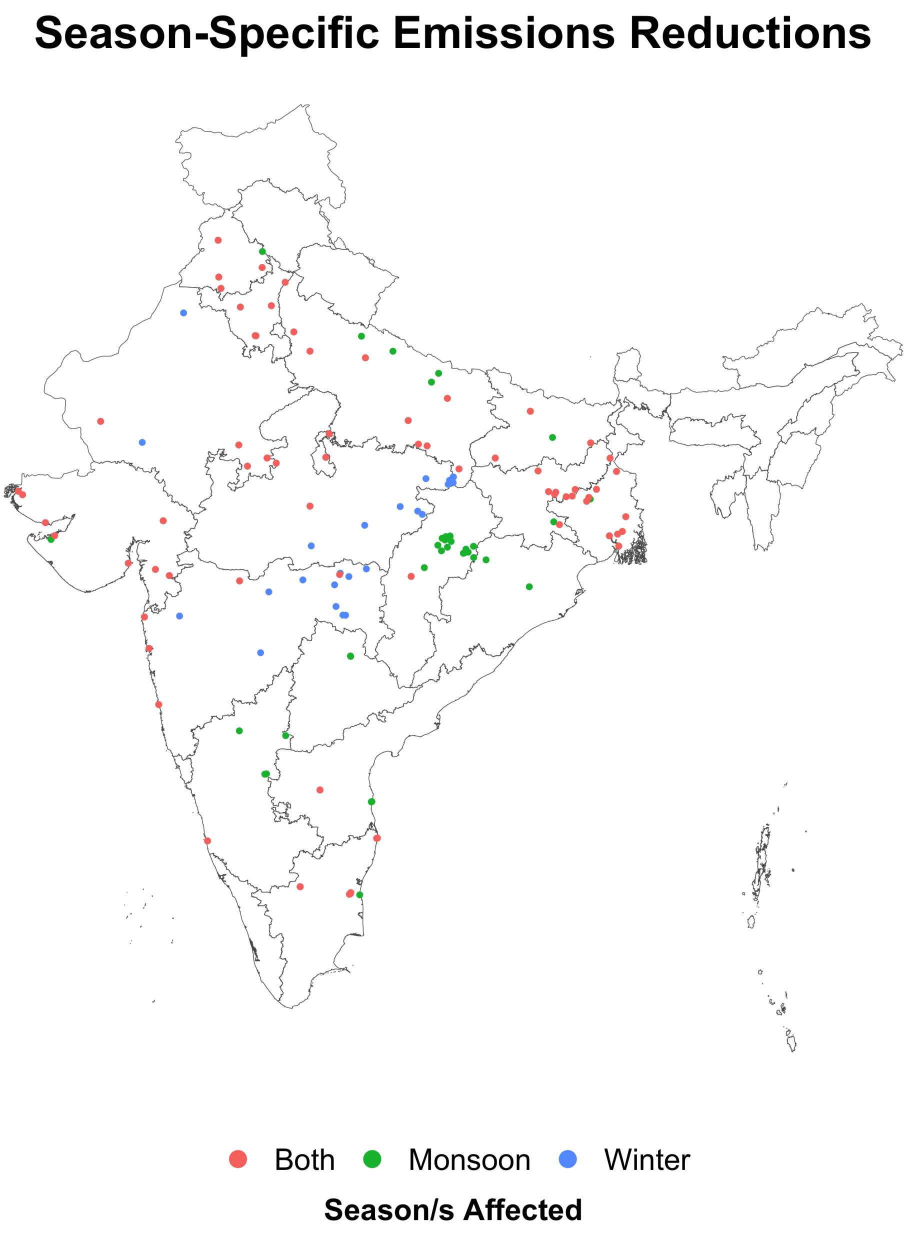

Fig. S16. Season and station-specific emissions reduction that maximizes total social benefits from only affecting 10% of total annual generation. Power stations whose emissions primarily result in wheat loss are selected for eliminating emissions in the winter (marked in blue), stations whose emissions primarily damage the rice crop are selected for monsoon emissions control (marked in green), while several stations that impact both crops (or have extremely high mortality damage intensity that is season-invariant) are selected in both seasons (marked in red). All generation and crop data from 2019.

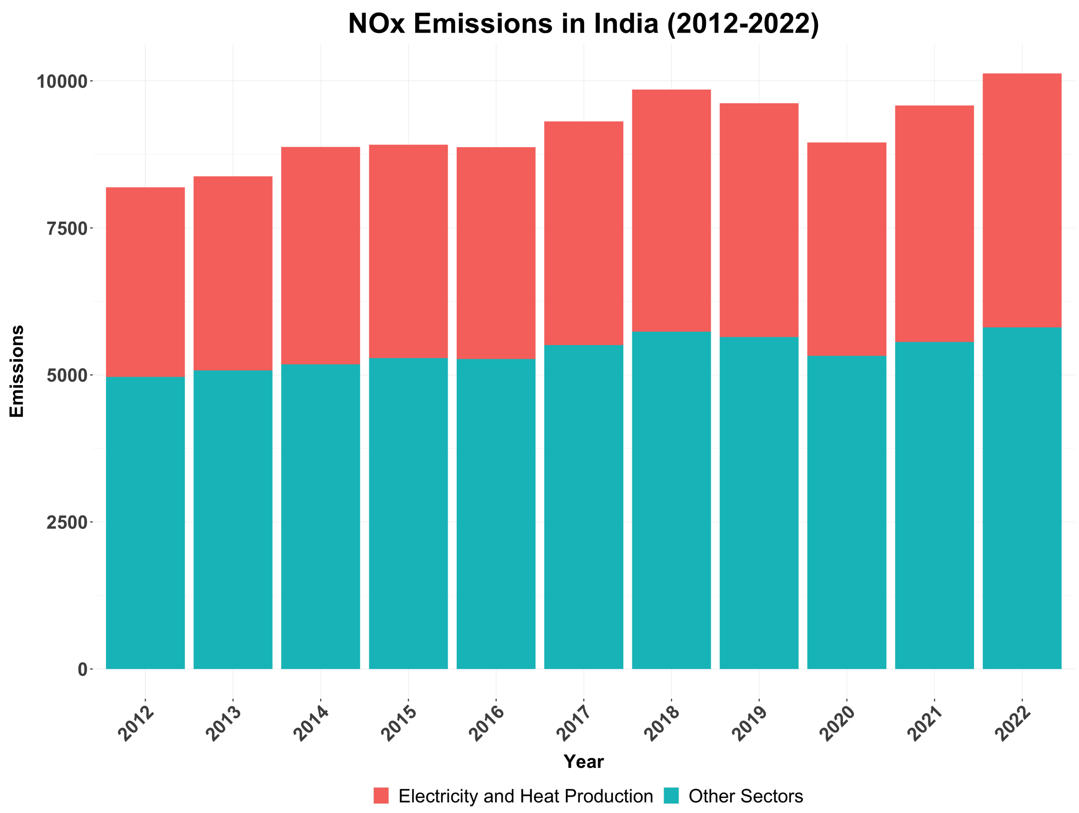

Fig. S17. Contribution of electricity and heat production sector to total anthropogenic NO_x emissions between 2012-2022, using data from the Emissions Database for Global Atmospheric Research (EDGAR). Electricity and heat production emissions, of which coal-fired electricity generation is a large share, have constituted 30-40% of total anthropogenic NO_x emissions in the country.

Table S1: Regression results for the monsoon season, as shown in Figure 1A. Separate models are estimated for exposure occurring in each distance category. The table below shows coefficients, standard errors, t-statistics and p-values for the five coal generation exposure variables in each model. Coefficients with p-values below 0.05 are considered statistically significant at the 95% level and are referred to as 'significant' in this study. Standard errors are clustered at the state-season level. In the underlying data, exposure is estimated in GWh and NO_2 concentrations in mol/m^2. The coefficients and standard errors are multiplied by 10^9 for legibility, and thereby correspond to the change in NO_2 concentration measured in $\mu mol/m^2$ associated with a 1 TWh change in exposure.

Regression Results (Monsoon)

Model	Exposure Direction	Coefficient	Std. Error	T-statistic	P-value
0-10 km	Upwind	29.17	3.53	8.26	0.00
0-10 km	Almost Upwind	24.20	2.61	9.28	0.00
0-10 km	Crosswind	13.15	2.28	5.76	0.00
0-10 km	Almost Downwind	4.68	2.98	1.57	0.12
0-10 km	Downwind	1.91	3.83	0.50	0.62
10-20 km	Upwind	22.06	2.90	7.62	0.00
10-20 km	Almost Upwind	14.15	1.89	7.49	0.00
10-20 km	Crosswind	5.29	0.92	5.73	0.00
10-20 km	Almost Downwind	0.13	1.74	0.07	0.94
10-20 km	Downwind	-3.37	2.89	-1.17	0.25
20-30 km	Upwind	14.95	2.86	5.23	0.00
20-30 km	Almost Upwind	8.07	1.12	7.20	0.00
20-30 km	Crosswind	2.70	0.75	3.59	0.00
20-30 km	Almost Downwind	-0.47	1.18	-0.40	0.69
20-30 km	Downwind	-3.89	2.34	-1.66	0.10
30-40 km	Upwind	11.52	2.60	4.44	0.00
30-40 km	Almost Upwind	5.48	1.11	4.96	0.00
30-40 km	Crosswind	1.14	0.77	1.48	0.14
30-40 km	Almost Downwind	-0.48	0.83	-0.58	0.56
30-40 km	Downwind	-3.96	2.27	-1.74	0.08
40-50 km	Upwind	7.74	1.94	3.98	0.00
40-50 km	Almost Upwind	3.83	1.05	3.63	0.00
40-50 km	Crosswind	0.55	0.70	0.79	0.43
40-50 km	Almost Downwind	0.37	0.66	0.56	0.58
40-50 km	Downwind	-3.54	2.06	-1.72	0.09
50-60 km	Upwind	5.69	1.65	3.44	0.00
50-60 km	Almost Upwind	2.57	0.90	2.85	0.01
50-60 km	Crosswind	1.33	0.74	1.81	0.07
50-60 km	Almost Downwind	0.53	0.53	1.00	0.32
50-60 km	Downwind	-2.82	1.59	-1.77	0.08
60-70 km	Upwind	5.63	1.85	3.04	0.00
60-70 km	Almost Upwind	1.80	0.80	2.24	0.03
60-70 km	Crosswind	1.56	0.88	1.77	0.08
60-70 km	Almost Downwind	-0.18	0.53	-0.35	0.73
60-70 km	Downwind	-2.27	1.32	-1.72	0.09
70-80 km	Upwind	3.10	1.48	2.10	0.04
70-80 km	Almost Upwind	2.19	0.77	2.82	0.01
70-80 km	Crosswind	0.32	0.66	0.49	0.63
70-80 km	Almost Downwind	-0.54	0.60	-0.90	0.37
70-80 km	Downwind	-0.85	1.00	-0.85	0.40
80-90 km	Upwind	2.82	0.88	3.20	0.00
80-90 km	Almost Upwind	1.44	0.62	2.31	0.02
80-90 km	Crosswind	-0.36	0.56	-0.65	0.51
80-90 km	Almost Downwind	0.01	0.47	0.01	0.99
80-90 km	Downwind	-1.37	0.92	-1.50	0.14
90-100 km	Upwind	1.49	0.68	2.18	0.03
90-100 km	Almost Upwind	0.85	0.50	1.72	0.09
90-100 km	Crosswind	-0.23	0.57	-0.40	0.69
90-100 km	Almost Downwind	-0.30	0.47	-0.64	0.53
90-100 km	Downwind	-1.34	0.91	-1.47	0.14

Table S2: Regression results for the winter season, as shown in Figure 1B. Separate models are estimated for exposure occurring in each distance category. The table below shows coefficients, standard errors, t-statistics and p-values for the five coal generation exposure variables in each model. Coefficients with p-values below 0.05 are considered statistically significant at the 95% level and are referred to as 'significant' in this study. Standard errors are clustered at the state-season level. In the underlying data, exposure is estimated in GWh and NO_2 concentrations in mol/m^2. The coefficients and standard errors are multiplied by 10^9 for legibility, and thereby correspond to the change in NO_2 concentration measured in $\mu mol/m^2$ associated with a 1 TWh change in exposure.

Regression Results (Winter)

Model	Exposure Direction	Coefficient	Std. Error	T-statistic	P-value
0-10 km	Upwind	30.28	3.23	9.39	0.00
0-10 km	Almost Upwind	24.74	2.50	9.89	0.00
0-10 km	Crosswind	16.67	2.26	7.37	0.00
0-10 km	Almost Downwind	11.18	2.36	4.74	0.00
0-10 km	Downwind	11.40	2.76	4.13	0.00
10-20 km	Upwind	20.36	2.59	7.86	0.00
10-20 km	Almost Upwind	12.18	1.49	8.18	0.00
10-20 km	Crosswind	4.82	1.30	3.72	0.00
10-20 km	Almost Downwind	5.30	1.36	3.89	0.00
10-20 km	Downwind	4.32	2.01	2.15	0.03
20-30 km	Upwind	12.59	2.28	5.53	0.00
20-30 km	Almost Upwind	6.77	1.02	6.62	0.00
20-30 km	Crosswind	1.60	0.74	2.15	0.03
20-30 km	Almost Downwind	2.35	0.95	2.47	0.02
20-30 km	Downwind	2.33	1.19	1.96	0.05
30-40 km	Upwind	10.08	2.02	4.99	0.00
30-40 km	Almost Upwind	4.75	0.88	5.40	0.00
30-40 km	Crosswind	0.67	0.62	1.08	0.28
30-40 km	Almost Downwind	1.30	0.82	1.58	0.12
30-40 km	Downwind	1.22	1.11	1.11	0.27
40-50 km	Upwind	8.05	1.85	4.36	0.00
40-50 km	Almost Upwind	2.92	0.92	3.20	0.00
40-50 km	Crosswind	0.27	0.71	0.39	0.70
40-50 km	Almost Downwind	1.17	0.68	1.72	0.09
40-50 km	Downwind	1.59	1.24	1.29	0.20
50-60 km	Upwind	7.12	1.45	4.90	0.00
50-60 km	Almost Upwind	1.11	0.77	1.44	0.15
50-60 km	Crosswind	0.51	0.50	1.01	0.31
50-60 km	Almost Downwind	0.53	0.56	0.94	0.35
50-60 km	Downwind	1.51	1.19	1.27	0.21
60-70 km	Upwind	6.32	1.31	4.82	0.00
60-70 km	Almost Upwind	1.02	0.75	1.36	0.18
60-70 km	Crosswind	-0.22	0.54	-0.41	0.68
60-70 km	Almost Downwind	0.09	0.58	0.16	0.88
60-70 km	Downwind	0.90	0.88	1.03	0.31
70-80 km	Upwind	4.66	1.27	3.69	0.00
70-80 km	Almost Upwind	0.31	0.67	0.46	0.65
70-80 km	Crosswind	0.12	0.57	0.22	0.83
70-80 km	Almost Downwind	0.07	0.53	0.13	0.89
70-80 km	Downwind	0.34	0.82	0.42	0.68
80-90 km	Upwind	3.36	1.18	2.85	0.01
80-90 km	Almost Upwind	0.09	0.62	0.15	0.88
80-90 km	Crosswind	-0.36	0.55	-0.66	0.51
80-90 km	Almost Downwind	-0.13	0.50	-0.26	0.79
80-90 km	Downwind	0.35	0.91	0.39	0.70
90-100 km	Upwind	2.63	1.02	2.56	0.01
90-100 km	Almost Upwind	-0.06	0.58	-0.10	0.92
90-100 km	Crosswind	-0.39	0.49	-0.79	0.43
90-100 km	Almost Downwind	-0.54	0.53	-1.02	0.31
90-100 km	Downwind	-0.20	0.80	-0.24	0.81

Table S3: Evaluation parameters for the NO_2 attribution models for the monsoon crop season. The adjusted R^2 in Column 2 measures how much of the total variation in seasonal NO_2 concentrations explained by the model (which includes the five generation exposure variables along with two meteorological variables - mean seasonal temperature and precipitation). The within-R^2 in Column 3 measures how much of the residual variation after excluding the fixed-effects for point and state-season is explained by the model. Column 4 shows the mean-squared error of each model. Columns 5 and 6 show the total number of observations used to fit each model and the total number of observations available in that distance-category. We do not use all available observations because we exclude points that are exposed to multiple coal power stations in that distance category to prevent over-estimating the effect of increased exposure to coal generation on concentrations at a point.

Model (1)	Adj. R^2 (2)	Within R^2 (3)	MSE ($\mu mol/m^2$) (4)	Obs. (5)	Total Obs. (6)
$\leq$10 km	0.87	0.16	11.2	126,256	179,378
10-20 km	0.85	0.11	8.2	332,340	532,256
20-30 km	0.85	0.07	6.7	532,123	884,268
30-40 km	0.87	0.06	5.7	721,607	1,203,506
40-50 km	0.85	0.03	5.4	866,139	1,528,742
50-60 km	0.87	0.02	5.2	1,036,220	1,845,272
60-70 km	0.87	0.02	5.1	1,154,604	2,167,599
70-80 km	0.87	0.01	5.09	1,282,054	2,512,760
80-90 km	0.86	0.01	4.69	1,379,712	2,807,358
90-100 km	0.87	0.0004	4.37	1,466,199	3,083,502

Table S4: Evaluation parameters for the NO_2 attribution models for the winter crop season. The adjusted R^2 in Column 2 measures how much of the total variation in seasonal NO_2 concentrations explained by the model (which includes the five generation exposure variables along with two meteorological variables - mean seasonal temperature and precipitation). The within-R^2 in Column 3 measures how much of the residual variation after excluding the fixed-effects for point and state-season is explained by the model. Column 4 shows the mean-squared error of each model. Columns 5 and 6 show the total number of observations used to fit each model and the total number of observations available in that distance-category. We do not use all available observations because we exclude points that are exposed to multiple coal power stations in that distance category to prevent over-estimating the effect of increased exposure to coal generation on concentrations at a point.

Model (1)	Adj. R^2 (2)	Within R^2 (3)	MSE ($\mu mol/m^2$) (4)	Obs. (5)	Total Obs. (6)
≤10 km	0.95	0.2	10.6	126,402	179,663
10-20 km	0.95	0.09	8.3	332,932	533,502
20-30 km	0.95	0.05	6.8	533,198	885,968
30-40 km	0.95	0.03	6.1	722,525	1,205,073
40-50 km	0.95	0.02	5.8	867,136	1,530,386
50-60 km	0.95	0.02	5.5	1,037,155	1,847,228
60-70 km	0.95	0.02	5.3	1,155,690	2,169,933
70-80 km	0.95	0.01	5	1,283,665	2,515,576
80-90 km	0.95	0.01	4.9	1,381,396	2,810,234
90-100 km	0.95	0.01	4.8	1,467,892	3,087,207

Table S5: Percentage of observed mean monsoon NO_2 concentrations in each state attributable to coal emissions using data from 2019. 95% prediction intervals in parentheses are computed using a cluster bootstrap. States with high levels of coal-fired electricity generation such as Chhattisgarh, Jharkhand, Tamil Nadu, and West Bengal have the highest fractions of coal-attributable NO_2.

Coal-attributable Monsoon NO2

State	Attributable NO2 (%)
Andhra Pradesh	10.28 (7.73-12.2)
Assam	4.62 (3.82-5.32)
Bihar	6.63 (5.06-7.43)
Chhattisgarh	19.12 (13.96-23.04)
Gujarat	4.66 (3.49-5.63)
Haryana	9.45 (7.67-10.8)
Jharkhand	14.41 (10.59-17.31)
Karnataka	3.64 (2.79-4.32)
Madhya Pradesh	8.57 (6.24-10.33)
Maharashtra	9.77 (7.79-11.25)
Odisha	7.52 (4.77-9.56)
Punjab	6.35 (5.05-7.32)
Rajasthan	7.54 (5.96-8.75)
Tamil Nadu	14.46 (10.28-16.21)
Telangana	14.12 (10.24-17.15)
Uttar Pradesh	5.26 (3.84-6.35)
West Bengal	12.71 (9.37-15.3)

Table S6: Percentage of observed mean winter NO_2 concentrations in each state attributable to coal emissions using data from 2019. 95% prediction intervals in parentheses are computed using a cluster bootstrap. As in the monsoon, states with high levels of coal-fired electricity generation have among the highest fractions of coal-attributable NO_2.

Coal-attributable Winter NO2

State	Attributable NO2 (%)
Andhra Pradesh	10.35 (8.06-13.12)
Assam	5.52 (4.63-6.35)
Bihar	3.92 (2.48-4.97)
Chhattisgarh	12.54 (7.74-15.25)
Gujarat	5.41 (3.76-6.18)
Haryana	11.24 (8.96-13.16)
Jharkhand	8.98 (5.04-11.79)
Karnataka	8.76 (6.06-10.02)
Madhya Pradesh	8.68 (5.66-10.63)
Maharashtra	7.68 (6.67-10.4)
Odisha	5.19 (3.12-6.92)
Punjab	5.42 (4.15-6.4)
Rajasthan	10.35 (8.31-13.66)
Tamil Nadu	16.28 (12.3-19.36)
Telangana	13.67 (9.68-16.77)
Uttar Pradesh	3.24 (2.15-4.01)
West Bengal	11.37 (7.93-13.27)

Table S7: Percentage of cropland studied (i.e. within 100 km of operational coal power stations) across different categories of expected yield gain from removing coal-attributable NO_2 concentrations. We focus on the top three states for the monsoon rice and winter wheat crop in terms of total output. For the rice crop, West Bengal is expected to see the greatest benefit across the largest fraction of cropland studied, with approximately 5.73% (4.04-6.95%) of cropland expected to see yield gains of between 5 and 10%. For the wheat crop, Madhya Pradesh is expected to see gains of between 5 and 10% on 5.98% (4.66-6.42%) of cropland and more than 10% on 11.98% (9.32-13.76%) of cropland. The intervals in parentheses are the 95% prediction interval estimated using a cluster bootstrap that includes uncertainty in the NO2-NIRv coefficient.

Cropland Fraction by Yield Gain (Monsoon)		Cropland Fraction by Yield Gain (Winter)	
Yield Gain	% Cropland Studied	Yield Gain	% Cropland Studied
Punjab		Madhya Pradesh	
1-5%	16.64 (13.5-19.74)	1-5%	19.57 (15.18-24.07)
5-10%	0.99 (0.6-1.44)	5-10%	5.98 (4.66-6.42)
<1%	82.32 (79.11-85.49)	<1%	62.48 (55.89-70.75)
>10%	0.05 (0.03-0.18)	>10%	11.98 (9.32-13.76)
Uttar Pradesh		Punjab	
1-5%	14.37 (11.91-16.67)	1-5%	18.33 (14.52-22.04)
5-10%	1.4 (1.04-1.76)	5-10%	1.8 (1.34-2.35)
<1%	83.73 (80.94-86.63)	<1%	79.57 (75.74-83.39)
>10%	0.49 (0.35-0.71)	>10%	0.3 (0.21-0.72)
West Bengal		Uttar Pradesh	
1-5%	33.67 (25.89-44.22)	1-5%	16.59 (13.04-19.63)
5-10%	5.73 (4.04-6.95)	5-10%	1.59 (1.29-2.24)
<1%	58.94 (47.59-68.26)	<1%	80.17 (76.24-84.06)
>10%	1.66 (1.03-2.33)	>10%	1.65 (1.3-2.12)

Table S8

Comparison of Mortality and Crop Damages (1/4)

Power Station	Mortality Damage ($ millions/year)	Wheat Damage ($ millions/year)	Rice Damage ($ millions/year)	Total Crop Damage ($ millions/year)
AKALTARA TPS	26.97 (23.38-29.53)	0.63 (0.5-0.73)	8.01 (6.58-9.07)	8.64 (7.08-9.79)
AKRIMOTA LIG TPS	18.57 (16.08-20.52)	0.01 (0-0.02)	0.02 (0.02-0.02)	0.03 (0.02-0.04)
AMARAVATI TPS	7.64 (6.62-8.37)	0.17 (0.13-0.2)	1.12 (0.88-1.3)	1.29 (1.02-1.5)
AMARKANTAK EXT TPS	4.69 (4.07-5.13)	0.72 (0.59-0.82)	0.4 (0.32-0.46)	1.12 (0.91-1.28)
ANPARA C TPS	26.75 (23.3-29.81)	3.08 (2.13-3.8)	1.63 (1.12-2.01)	4.71 (3.25-5.8)
ANPARA TPS	55.51 (48.37-61.9)	8.16 (5.61-10.07)	3.87 (2.64-4.78)	12.02 (8.25-14.84)
ANUPPUR TPP	18.28 (15.86-20.12)	2.27 (1.86-2.6)	1.39 (1.1-1.62)	3.66 (2.96-4.22)
BAKRESWAR TPS	29.24 (25.72-31.48)	1.64 (1.03-2.12)	9.68 (7.48-11.34)	11.32 (8.51-13.46)
BANDAKHAR TPP	5.16 (4.48-5.63)	0.09 (0.06-0.11)	0.8 (0.65-0.91)	0.89 (0.71-1.02)
BANDEL TPS	8.48 (7.49-9.08)	0.15 (0.12-0.18)	1.65 (1.36-1.88)	1.8 (1.48-2.06)
BARADARHA TPS	19.93 (17.3-21.88)	0.59 (0.49-0.66)	3.76 (3.08-4.25)	4.34 (3.57-4.92)
BARAUNI TPS	0.39 (0.34-0.43)	0 (0-0)	0.3 (0.23-0.36)	0.3 (0.23-0.36)
BARKHERA TPS	0.23 (0.2-0.26)	0 (0-0)	0.1 (0.08-0.11)	0.1 (0.08-0.11)
BELLARY TPS	13.31 (11.55-14.55)	0.29 (0.22-0.34)	1.68 (1.31-1.95)	1.97 (1.53-2.29)
BHAVNAGAR CFBC TPP	26.17 (22.68-28.73)	0.19 (0.14-0.23)	0.04 (0.03-0.05)	0.23 (0.17-0.28)
BHILAI TPS	13.38 (11.59-14.6)	0.37 (0.28-0.44)	2.52 (2.03-2.9)	2.88 (2.32-3.33)
BHUSAWAL TPS	34.95 (30.34-38.37)	2.77 (1.95-3.39)	0.61 (0.45-0.72)	3.38 (2.4-4.11)
BINA TPS	8.41 (7.27-9.24)	2.79 (2.06-3.35)	0.74 (0.57-0.87)	3.53 (2.63-4.23)
BINJKOTE TPP	7.3 (6.34-7.96)	0.1 (0.08-0.11)	1.94 (1.56-2.23)	2.04 (1.64-2.34)
BOKARO TPS A EXP	9.31 (8.13-10.14)	1.23 (0.91-1.47)	0.42 (0.27-0.54)	1.65 (1.17-2.01)
BONGAIGAON TPP	0.55 (0.47-0.62)	0.05 (0.04-0.06)	2.33 (1.91-2.64)	2.37 (1.94-2.7)
BUDGE BUDGE TPS	149.06 (131.6-159.4)	0.55 (0.38-0.68)	7.95 (6.19-9.29)	8.51 (6.57-9.97)
BUTIBORI TPP	0.16 (0.14-0.17)	0.28 (0.21-0.33)	0 (0-0)	0.28 (0.21-0.33)
CHAKABURA TPP	0.49 (0.42-0.53)	0.02 (0.01-0.02)	0.12 (0.09-0.13)	0.13 (0.1-0.15)
CHANDRAPUR MAHARASHTRA STPS	48.17 (41.8-52.64)	7.25 (5.72-8.5)	1.59 (1.19-1.88)	8.84 (6.91-10.39)
CHANDRAPURA DVC TPS	12.23 (10.67-13.32)	1.11 (0.82-1.32)	1.76 (1.31-2.11)	2.87 (2.13-3.43)
CHHABRA TPP	46.13 (40.01-51.62)	12.53 (9.53-14.87)	2.68 (2.07-3.12)	15.21 (11.6-17.99)
DADRI NCTPP	40.5 (35.54-45.77)	15.4 (11.34-18.47)	3.84 (2.82-4.6)	19.24 (14.17-23.07)
DAHANU TPS	19.65 (17.07-21.44)	0.27 (0.23-0.31)	0.08 (0.06-0.09)	0.35 (0.29-0.4)
DAMODARAM SANJEEVAIAH TPS	29.1 (25.36-31.56)	0 (0-0)	1.51 (1.23-1.73)	1.51 (1.23-1.73)
DERANG TPP	12.23 (10.63-13.29)	0 (0-0)	1.24 (0.94-1.47)	1.24 (0.94-1.47)
DHARIWAL TPP	8.25 (7.16-9.02)	0.85 (0.69-0.99)	0.48 (0.37-0.55)	1.33 (1.06-1.54)
DURGAPUR STEEL TPS	28.3 (24.92-30.39)	1.13 (0.61-1.53)	6.76 (5.28-7.85)	7.89 (5.89-9.38)
DURGAPUR TPS	2.27 (2-2.43)	0.15 (0.08-0.2)	0.53 (0.38-0.64)	0.68 (0.46-0.85)
FARAKKA STPS	63.16 (55.45-68.49)	4.36 (2.73-5.63)	10.69 (7.95-12.71)	15.05 (10.69-18.34)
GANDHI NAGAR TPS	6.98 (6.01-7.85)	2.1 (1.6-2.49)	0.04 (0.03-0.04)	2.14 (1.62-2.53)

Table S8

Comparison of Mortality and Crop Damages (2/4)

Power Station	Mortality Damage ($ millions/year)	Wheat Damage ($ millions/year)	Rice Damage ($ millions/year)	Total Crop Damage ($ millions/year)
GH TPS LEH MOH	3.73 (3.27-4.05)	0.56 (0.43-0.66)	1.72 (1.36-1.99)	2.27 (1.78-2.64)
GMR WARORA TPS	11 (9.53-12.04)	2.35 (1.92-2.69)	0.53 (0.42-0.61)	2.88 (2.34-3.3)
GOINDWAL SAHIB TPP	9.66 (8.45-10.56)	8.5 (6.89-9.81)	3.69 (2.94-4.28)	12.19 (9.82-14.09)
HALDIA TPP	104.32 (92.07-111.58)	0.29 (0.22-0.34)	4.89 (3.93-5.61)	5.18 (4.15-5.95)
HARDUAGANJ TPS	21.69 (18.99-24.54)	4.33 (3.33-5.09)	3.03 (2.4-3.5)	7.36 (5.73-8.59)
IB VALLEY TPS	9.51 (8.25-10.37)	0.03 (0-0.05)	4.7 (3.69-5.5)	4.73 (3.69-5.55)
INDIRA GANDHI STPP	17.99 (15.67-20.25)	17.97 (14.45-20.73)	4.46 (3.68-5.06)	22.43 (18.12-25.79)
ITPCL TPP	30.05 (26.33-32.23)	0 (0-0)	5.6 (4.2-6.65)	5.6 (4.2-6.65)
JALIPA KAPURDI TPP	147.24 (127.22-164.85)	0.36 (0.11-0.55)	0.1 (0.08-0.11)	0.46 (0.19-0.66)
JOJOBERA TPS	4.65 (4.07-5.05)	0.42 (0.33-0.49)	0.95 (0.68-1.16)	1.37 (1.01-1.65)
JSW RATNAGIRI TPP	38.54 (33.47-42.07)	0 (-0.11-0.09)	0.03 (0.01-0.05)	0.04 (-0.1-0.14)
KAHALGAON TPS	70.8 (61.83-77.45)	13.81 (10.49-16.28)	12.3 (9.16-14.78)	26.11 (19.65-31.06)
KAKATIYA TPS	10.33 (8.98-11.27)	0.08 (0.06-0.09)	0.82 (0.53-1.05)	0.89 (0.59-1.14)
KALISINDH TPS	32.31 (28.03-36.17)	9.07 (6.92-10.68)	0.56 (0.42-0.68)	9.63 (7.34-11.35)
KAMALANGA TPS	18.83 (16.38-20.41)	0 (0-0)	1.2 (0.87-1.45)	1.2 (0.87-1.45)
KASAIPALLI TPP	3.02 (2.61-3.31)	0.06 (0.04-0.08)	0.8 (0.63-0.93)	0.86 (0.67-1.01)
KAWAI TPS	36.39 (31.6-40.69)	9.56 (7.2-11.34)	0.69 (0.42-0.91)	10.26 (7.62-12.25)
KHAMBARKHERA TPS	0.22 (0.2-0.25)	0 (0-0)	0.05 (0.04-0.06)	0.05 (0.04-0.06)
KHAPARKHEDA TPS	29.68 (25.75-32.54)	4.36 (3.1-5.3)	0.66 (0.49-0.79)	5.02 (3.6-6.08)
KODARMA TPP	22.49 (19.62-24.72)	3.7 (2.62-4.53)	3.92 (3.08-4.55)	7.62 (5.7-9.08)
KOLAGHAT TPS	52.79 (46.6-56.45)	0.43 (0.31-0.52)	4.37 (3.5-5.02)	4.8 (3.81-5.54)
KORADI TPS	39.16 (33.98-42.88)	5.24 (3.72-6.38)	0.74 (0.55-0.87)	5.98 (4.27-7.25)
KORBA WEST TPS	22.24 (19.29-24.33)	0.59 (0.42-0.72)	4.98 (3.85-5.83)	5.58 (4.27-6.55)
KORBA STPS	53.95 (46.84-59.13)	1.29 (0.93-1.57)	10.08 (7.96-11.65)	11.37 (8.89-13.22)
KOTA TPS	32.67 (28.27-36.81)	6.28 (4.42-7.67)	0.18 (0.12-0.22)	6.46 (4.54-7.9)
KOTHAGUDEM TPS NEW	18.01 (15.66-19.61)	0.03 (0.02-0.04)	2.19 (1.42-2.8)	2.22 (1.45-2.84)
KUDGI STPP	24.82 (21.53-27.16)	0.7 (0.47-0.87)	0.51 (0.41-0.58)	1.21 (0.89-1.46)
KUNDARKI TPS	0.38 (0.33-0.43)	0 (0-0)	0.07 (0.06-0.08)	0.07 (0.06-0.08)
KUTCH LIG TPS	18.38 (15.91-20.31)	0.03 (0.02-0.04)	0.02 (0.02-0.03)	0.06 (0.04-0.07)
LALITPUR TPS	25.17 (21.86-28.17)	1.51 (1.18-1.75)	4.09 (3.35-4.65)	5.6 (4.53-6.41)
LARA TPP	4.34 (3.76-4.73)	0.02 (0.01-0.02)	2.08 (1.74-2.35)	2.1 (1.75-2.37)
MAHADEV PRASAD STPP	6.39 (5.58-6.94)	0.61 (0.47-0.72)	1.94 (1.42-2.37)	2.55 (1.9-3.09)
MAHATMA GANDHI TPS	20.9 (18.21-23.55)	16.9 (13.61-19.52)	5.35 (4.38-6.09)	22.25 (17.99-25.61)
MAITHON RB TPP	23.77 (20.84-25.72)	2.06 (1.4-2.55)	6.64 (4.91-7.97)	8.71 (6.31-10.52)
MARWA TPS	14.11 (12.25-15.47)	0.35 (0.28-0.41)	3.43 (2.82-3.87)	3.78 (3.1-4.28)
MAUDA TPS	35.37 (30.7-38.76)	6.01 (4.5-7.18)	1.45 (1.12-1.7)	7.47 (5.62-8.88)

Table S8

Power Station	Mortality Damage ($ millions/year)	Wheat Damage ($ millions/year)	Rice Damage ($ millions/year)	Total Crop Damage ($ millions/year)
MEJA STPP	3.55 (3.1-4.07)	0.12 (0.1-0.14)	0.51 (0.41-0.59)	0.63 (0.5-0.73)
MEJIA TPS	58.63 (51.66-62.86)	2.44 (1.44-3.23)	17.23 (13.46-20.04)	19.66 (14.91-23.26)
METTUR TPS	59.23 (51.91-63.5)	0.01 (0-0.02)	2.51 (1.94-2.93)	2.52 (1.94-2.95)
METTUR TPS II	44.87 (39.32-48.11)	0.01 (0-0.01)	1.91 (1.48-2.23)	1.91 (1.48-2.24)
MUNDRA UMTPP	207.75 (180.17-228.54)	1.92 (1.37-2.39)	1.64 (1.12-2.01)	3.56 (2.49-4.4)
MUTHIARA TPP	3.75 (3.29-4.03)	0 (0-0)	1.16 (0.96-1.31)	1.16 (0.96-1.31)
MUZAFFARPUR TPS	12.16 (10.67-13.65)	3.7 (2.88-4.36)	2.57 (2.08-2.94)	6.27 (4.97-7.3)
NABI NAGAR TPP	12.53 (10.94-14.08)	4.2 (3.23-4.94)	3.67 (2.93-4.23)	7.87 (6.16-9.17)
NASIK TPS	9.37 (8.14-10.22)	0.48 (0.39-0.56)	0.22 (0.18-0.25)	0.71 (0.58-0.81)
NAWAPARA TPP	6.75 (5.85-7.36)	0.19 (0.16-0.22)	0.55 (0.42-0.64)	0.74 (0.58-0.85)
NEYVELI EXT TPS	158.66 (139.08-170.06)	0 (0-0)	3.12 (2.43-3.67)	3.12 (2.43-3.67)
NEYVELI TPS Z	60.7 (53.22-65.05)	0 (0-0)	1.56 (1.24-1.79)	1.56 (1.24-1.79)
NEYVELI TPS II	496.8 (435.55-532.34)	0 (0-0)	9.35 (7.32-10.91)	9.35 (7.32-10.91)
NEYVELI TPS II EXP	82.42 (72.26-88.32)	0 (0-0)	1.8 (1.4-2.1)	1.8 (1.4-2.1)
NIGRI TPP	19.13 (16.66-21.32)	3.73 (2.74-4.5)	1.17 (0.88-1.4)	4.9 (3.62-5.91)
NORTH CHENNAI TPS	46.8 (40.94-50.38)	0 (0-0)	2.73 (2.17-3.17)	2.73 (2.17-3.17)
OBRA TPS	14.23 (12.4-15.94)	2.29 (1.59-2.83)	1.18 (0.85-1.42)	3.47 (2.45-4.25)
PAINAMPURAM TPP	39.03 (34.02-42.33)	0 (0-0)	2.33 (1.91-2.65)	2.33 (1.91-2.65)
PANIPAT TPS	16.66 (14.54-18.59)	5.32 (4.11-6.24)	4.16 (3.38-4.74)	9.48 (7.49-10.98)
PARAS TPS	8.77 (7.6-9.6)	0.78 (0.56-0.95)	0.18 (0.14-0.21)	0.96 (0.7-1.15)
PARICHHA TPS	17.87 (15.53-20.02)	7.99 (6.19-9.37)	2.25 (1.78-2.61)	10.24 (7.98-11.98)
PARLI TPS	5.77 (5.01-6.29)	0.16 (0.12-0.19)	0.48 (0.38-0.56)	0.64 (0.5-0.75)
PATHADI TPP	11.07 (9.61-12.11)	0.33 (0.26-0.39)	2.2 (1.82-2.49)	2.54 (2.08-2.88)
PRAYAGRAJ TPP	32.5 (28.35-36.98)	10.93 (8.37-12.91)	5.15 (4.09-5.94)	16.08 (12.47-18.85)
RAGHUNATHPUR TPP	23.28 (20.47-25.05)	0.93 (0.61-1.16)	5.34 (4.16-6.25)	6.27 (4.77-7.42)
RAICHUR TPS	33.09 (28.73-36.16)	0.61 (0.5-0.7)	1.77 (1.36-2.08)	2.38 (1.85-2.78)
RAIKHEDA TPP	17.64 (15.3-19.32)	0.33 (0.25-0.39)	5.92 (4.71-6.81)	6.25 (4.97-7.2)
RAJIV GANDHI TPS	8.15 (7.12-9.08)	2.97 (2.29-3.5)	4.34 (3.31-5.14)	7.31 (5.6-8.64)
RAJPURA TPP	42.13 (36.71-46.56)	25.96 (20.97-29.97)	20.96 (17.53-23.46)	46.92 (38.49-53.42)
RAMAGUNDEM B TPS	1.48 (1.28-1.61)	0 (0-0.01)	0.06 (0.04-0.07)	0.06 (0.05-0.08)
RAMAGUNDEM STPS	55.14 (47.91-60.14)	0.15 (0.02-0.25)	4.25 (3.27-5.01)	4.4 (3.3-5.26)
RATIJA TPS	1.66 (1.43-1.82)	0.05 (0.04-0.07)	0.33 (0.27-0.37)	0.38 (0.3-0.44)
RAYALASEEMA TPS	39.43 (34.32-42.88)	0 (0-0)	2.49 (1.96-2.9)	2.49 (1.96-2.9)
RIHAND STPS	72.58 (63.19-80.53)	8.1 (5.65-9.93)	4.95 (3.29-6.2)	13.05 (8.93-16.14)
ROPAR TPS	2.16 (1.86-2.39)	0 (0-0)	1.38 (1.09-1.58)	1.38 (1.09-1.58)
ROSA TPP Ph I	35.54 (31.1-40.11)	1.65 (1.28-1.93)	5.64 (4.48-6.52)	7.29 (5.76-8.46)

Table S8

Comparison of Mortality and Crop Damages (4/4)

Power Station	Mortality Damage ($ millions/year)	Wheat Damage ($ millions/year)	Rice Damage ($ millions/year)	Total Crop Damage ($ millions/year)
SAGARDIGHI TPS	28.64 (25.2-30.9)	1.17 (0.85-1.42)	9.43 (7.75-10.71)	10.59 (8.59-12.13)
SALAYA TPP	27.43 (23.78-30.15)	0 (0-0)	0.75 (0.63-0.85)	0.75 (0.63-0.85)
SANJAY GANDHI TPS	16.53 (14.33-18.17)	3.64 (2.63-4.42)	0.74 (0.56-0.88)	4.38 (3.2-5.29)
SANTALDIH TPS	15.28 (13.39-16.52)	0.92 (0.62-1.14)	3.7 (2.83-4.36)	4.62 (3.46-5.5)
SASAN UMTPP	94.39 (82.08-104.3)	12.89 (9.32-15.63)	6.59 (4.39-8.24)	19.48 (13.71-23.88)
SATPURA TPS	19.21 (16.62-21.1)	5.64 (4.15-6.74)	0.39 (0.3-0.46)	6.03 (4.45-7.2)
SEIONI TPP	8.77 (7.61-9.66)	2.18 (1.68-2.58)	0.4 (0.32-0.47)	2.58 (2.01-3.05)
SGPL TPP	37.57 (32.74-40.74)	0 (0-0)	2.28 (1.86-2.59)	2.28 (1.86-2.59)
SIKKA REP TPS	23.13 (20.05-25.42)	0.31 (0.2-0.39)	0.19 (0.16-0.22)	0.5 (0.36-0.61)
SINGARENI TPP	27.04 (23.51-29.48)	0.18 (0.07-0.26)	1.75 (1.3-2.1)	1.93 (1.37-2.36)
SINGRAULI STPS	41.82 (36.39-46.45)	6.09 (4.29-7.43)	2.84 (1.9-3.56)	8.93 (6.18-11)
SIPAT STPS	72.98 (63.33-80)	2.41 (1.87-2.82)	15.5 (12.89-17.44)	17.91 (14.76-20.26)
SOUTHERN REPL TPS	6.95 (6.13-7.43)	0.01 (0.01-0.02)	0.57 (0.44-0.67)	0.58 (0.45-0.68)
SURATGARH TPS	14.91 (12.93-16.54)	3.95 (2.33-5.14)	-0.02 (-0.24-0.2)	3.93 (2.09-5.34)
SURAT LIG TPS	144.9 (125.66-159.21)	1.42 (1.15-1.64)	0.34 (0.28-0.38)	1.76 (1.42-2.03)
SVPL TPP	0.4 (0.34-0.44)	0 (0-0)	0.16 (0.13-0.18)	0.16 (0.13-0.18)
TALCHER STPS	58.12 (50.6-63.12)	0 (0-0)	4.1 (3.04-4.93)	4.1 (3.04-4.93)
TALWANDI SABO TPP	45.51 (39.74-50.26)	24.91 (19.28-29.27)	17.47 (13.6-20.48)	42.38 (32.88-49.75)
TAMNAR TPP	27.16 (23.58-29.79)	0.44 (0.3-0.54)	3.82 (2.93-4.52)	4.26 (3.23-5.06)
TANDA TPS	8.06 (7.08-9.24)	3.94 (2.99-4.66)	2.16 (1.69-2.51)	6.1 (4.68-7.17)
TENUGHAT TPS	6.81 (5.94-7.42)	0.59 (0.44-0.7)	1.34 (0.99-1.61)	1.93 (1.44-2.31)
TIRORA TPS	75.11 (65.22-82.49)	12.82 (10.02-15.01)	2.25 (1.6-2.76)	15.07 (11.62-17.77)
TORANGALLU TPS SBU I	4.11 (3.57-4.5)	0.06 (0.04-0.07)	0.53 (0.41-0.61)	0.59 (0.46-0.68)
TROMBAY TPS	31.58 (27.44-34.41)	0.13 (0.08-0.17)	0.1 (0.08-0.11)	0.22 (0.16-0.28)
TUTICORIN JV TPP	5.08 (4.45-5.45)	0 (0-0)	1.09 (0.78-1.31)	1.09 (0.78-1.31)
TUTICORIN TPS	5.92 (5.19-6.35)	0 (0-0)	1.56 (1.22-1.81)	1.56 (1.22-1.81)
UCHPINDA TPP	5.54 (4.8-6.05)	0.13 (0.11-0.14)	1.37 (1.12-1.56)	1.5 (1.22-1.71)
UDUPI TPP	25.33 (22.02-27.6)	0 (0-0)	0 (0-0)	0 (0-0)
UKAI TPS	35.85 (31.09-39.31)	2.4 (1.89-2.81)	0.44 (0.35-0.5)	2.84 (2.24-3.31)
UNCHAHAR TPS	41.83 (36.42-47.86)	15.31 (11.72-18.08)	6.93 (5.5-8.02)	22.24 (17.22-26.1)
UTRAULA TPS	0.45 (0.4-0.51)	0 (0-0)	0.12 (0.09-0.13)	0.12 (0.09-0.13)
VALLUR TPP	38.77 (33.93-41.7)	0 (0-0)	1.51 (1.21-1.73)	1.51 (1.21-1.73)
VINDHYACHAL STPS	111.29 (96.86-123.51)	15.11 (10.88-18.31)	6.76 (4.52-8.46)	21.87 (15.39-26.78)
VIZAG TPP	5.57 (4.85-6.04)	0 (0-0)	0.23 (0.19-0.26)	0.23 (0.19-0.26)
WANAKBORI TPS	25.32 (21.93-27.86)	4.27 (3.22-5.07)	0.24 (0.19-0.28)	4.51 (3.41-5.34)
YAMUNA NAGAR TPS	22.07 (19.11-24.66)	7.49 (5.9-8.77)	3.61 (3.05-4.06)	11.11 (8.95-12.82)

Table S9

Comparison of Mortality and Crop Damage Intensity (1/4)

Power Station	Mortality Damage Intensity ($/GWh-year)	Wheat Damage Intensity ($/GWh-winter)	Rice Damage Intensity ($/GWh-monsoon)	Avg Crop Damage Intensity ($/GWh-year)
AKALTARA TPS	3066 (2658-3357)	709 (565-822)	6178 (5074-6991)	982 (805-1113)
AKRIMOTA LIG TPS	22501 (19480-24858)	60 (15-93)	205 (174-229)	39 (26-49)
AMARAVATI TPS	2509 (2174-2748)	3695 (2859-4342)	981 (773-1139)	425 (334-494)
AMARKANTAK EXT TPS	2784 (2415-3045)	2443 (2008-2794)	1430 (1144-1657)	663 (539-761)
ANPARA C TPS	3473 (3026-3871)	2558 (1770-3150)	1359 (933-1675)	612 (422-754)
ANPARA TPS	3033 (2642-3381)	2546 (1752-3142)	1331 (907-1644)	657 (451-811)
ANUPPUR TPP	2804 (2433-3087)	2115 (1738-2429)	1691 (1332-1969)	561 (454-648)
BAKRESWAR TPS	4260 (3747-4587)	1308 (820-1683)	10089 (7796-11817)	1650 (1240-1960)
BANDAKHAR TPP	2745 (2381-2995)	443 (296-556)	4637 (3778-5273)	474 (379-545)
BANDEL TPS	8003 (7061-8563)	711 (551-841)	9888 (8137-11247)	1701 (1393-1941)
BARADARHA TPS	3331 (2891-3656)	514 (429-582)	5066 (4159-5740)	725 (597-822)
BARAUNI TPS	2812 (2455-3115)	0 (0-0)	4052 (3062-4805)	2195 (1659-2603)
BARKHERA TPS	2563 (2229-2830)	0 (0-0)	5814 (4853-6544)	1091 (910-1228)
BELLARY TPS	3283 (2849-3590)	344 (265-404)	2866 (2236-3326)	485 (378-564)
BHAVNAGAR CFBC TPP	50160 (43477-55073)	1452 (1058-1747)	618 (467-733)	450 (331-541)
BHILAI TPS	4496 (3896-4908)	798 (612-944)	6293 (5091-7246)	970 (779-1120)
BHUSAWAL TPS	5697 (4947-6254)	2609 (1831-3189)	895 (663-1066)	551 (391-671)
BINA TPS	3227 (2792-3547)	6378 (4709-7671)	1664 (1277-1959)	1354 (1008-1622)
BINJKOTE TPP	2868 (2488-3127)	427 (347-492)	4282 (3446-4910)	800 (644-918)
BOKARO TPS A EXP	3217 (2809-3503)	2178 (1608-2611)	3192 (2029-4098)	569 (406-695)
BONGAIGAON TPP	205 (175-229)	100 (77-119)	4553 (3736-5174)	879 (720-1000)
BUDGE BUDGE TPS	25982 (22937-27782)	565 (393-698)	8192 (6375-9566)	1483 (1146-1738)
BUTIBORI TPP	1808 (1570-1979)	3225 (2473-3820)	0 (0-0)	3225 (2473-3820)
CHAKABURA TPP	2074 (1793-2270)	403 (271-505)	3958 (3144-4563)	563 (439-655)
CHANDRAPUR MAHARASHTRA STPS	2975 (2582-3251)	2280 (1799-2673)	817 (612-968)	546 (427-641)
CHANDRAPURA DVC TPS	3818 (3331-4161)	2251 (1667-2687)	4275 (3169-5108)	896 (664-1070)
CHHABRA TPP	4528 (3927-5067)	6868 (5225-8150)	1226 (949-1429)	1493 (1139-1766)
DADRI NCTPP	5495 (4822-6210)	9891 (7286-11863)	4184 (3080-5016)	2610 (1922-3130)
DAHANU TPS	5877 (5104-6412)	592 (489-678)	177 (148-198)	105 (87-120)
DAMODARAM SANJEEVAIAH TPS	4143 (3611-4493)	0 (0-0)	1516 (1230-1737)	215 (175-247)
DERANG TPP	2678 (2328-2910)	0 (0-0)	1861 (1413-2206)	272 (207-323)
DHARIWAL TPP	2848 (2471-3114)	2521 (2035-2916)	943 (732-1098)	459 (365-532)
DURGAPUR STEEL TPS	4453 (3921-4782)	994 (539-1346)	10031 (7834-11653)	1241 (927-1475)
DURGAPUR TPS	3825 (3372-4097)	943 (509-1281)	8311 (5914-10116)	1149 (774-1434)
FARAKKA STPS	4724 (4147-5122)	1740 (1090-2245)	5481 (4079-6518)	1126 (799-1372)
GANDHI NAGAR TPS	3544 (3048-3982)	4180 (3176-4951)	1145 (887-1336)	1084 (824-1283)

Table S9

Comparison of Mortality and Crop Damage Intensity (2/4)

Power Station	Mortality Damage Intensity ($/GWh-year)	Wheat Damage Intensity ($/GWh-winter)	Rice Damage Intensity ($/GWh-monsoon)	Avg Crop Damage Intensity ($/GWh-year)
GH TPS LEH MOH	3891 (3409-4230)	17367 (13283-20479)	13416 (10591-15525)	2373 (1859-2759)
GMR WARORA TPS	2626 (2276-2876)	3348 (2736-3834)	966 (770-1110)	687 (559-788)
GOINDWAL SAHIB TPP	4784 (4188-5229)	17251 (13971-19912)	11851 (9439-13756)	6039 (4866-6982)
HALDIA TPP	23297 (20562-24919)	476 (360-564)	6501 (5221-7453)	1157 (926-1329)
HARDUAGANJ TPS	6834 (5984-7733)	12132 (9340-14270)	5711 (4519-6583)	2320 (1807-2706)
IB VALLEY TPS	3434 (2979-3745)	53 (-2-95)	3402 (2671-3981)	1707 (1333-2004)
INDIRA GANDHI STPP	3862 (3363-4346)	13156 (10572-15174)	6908 (5702-7838)	4814 (3890-5535)
ITPCL TPP	4695 (4114-5037)	0 (0-0)	4045 (3036-4808)	875 (657-1040)
JALIPA KAPURDI TPP	25109 (21694-28112)	360 (114-546)	109 (89-124)	78 (33-112)
JOJOBERA TPS	3150 (2753-3421)	1713 (1343-2006)	4250 (3057-5169)	929 (686-1116)
JSW RATNAGIRI TPP	4863 (4223-5308)	3 (-89-70)	28 (11-41)	5 (-13-17)
KAHALGAON TPS	4331 (3782-4738)	5059 (3843-5966)	4571 (3404-5493)	1597 (1202-1900)
KAKATIYA TPS	2693 (2340-2936)	89 (67-108)	1163 (761-1489)	233 (155-297)
KALISINDH TPS	5463 (4739-6116)	6505 (4965-7661)	704 (527-844)	1628 (1241-1919)
KAMALANGA TPS	3226 (2807-3496)	0 (0-0)	2195 (1589-2661)	205 (149-249)
KASAIPALLI TPP	1946 (1683-2129)	407 (273-509)	3743 (2940-4349)	557 (433-651)
KAWAI TPS	4459 (3872-4986)	6416 (4830-7607)	526 (318-689)	1257 (934-1501)
KHAMBARKHERA TPS	2797 (2437-3094)	0 (0-0)	4941 (4080-5581)	661 (546-746)
KHAPARKHEDA TPS	4043 (3508-4433)	3021 (2152-3673)	795 (593-947)	684 (490-829)
KODARMA TPP	3436 (2997-3777)	3327 (2358-4075)	3873 (3040-4490)	1165 (871-1387)
KOLAGHAT TPS	15300 (13506-16360)	660 (475-802)	9357 (7495-10742)	1391 (1104-1606)
KORADI TPS	4206 (3650-4605)	2915 (2072-3548)	776 (580-922)	642 (459-779)
KORBA WEST TPS	2489 (2160-2724)	389 (278-476)	3471 (2682-4062)	624 (478-734)
KORBA STPS	2737 (2376-3000)	394 (285-480)	3804 (3006-4399)	577 (451-671)
KOTA TPS	4742 (4104-5342)	4465 (3144-5452)	212 (138-268)	938 (659-1146)
KOTHAGUDEM TPS NEW	2588 (2251-2818)	12 (9-14)	948 (616-1215)	319 (208-408)
KUDGI STPP	4464 (3872-4884)	362 (245-451)	2210 (1789-2520)	218 (160-262)
KUNDARKI TPS	3142 (2773-3571)	0 (0-0)	4944 (3947-5713)	583 (466-674)
KUTCH LIG TPS	26638 (23061-29437)	138 (87-181)	260 (219-293)	82 (59-101)
LALITPUR TPS	3698 (3210-4138)	8915 (6982-10367)	3343 (2739-3805)	822 (665-941)
LARA TPP	2926 (2539-3190)	292 (231-338)	5475 (4563-6181)	1417 (1180-1600)
MAHADEV PRASAD STPP	2271 (1985-2466)	1901 (1479-2246)	3848 (2814-4686)	908 (674-1098)
MAHATMA GANDHI TPS	3358 (2926-3785)	13087 (10537-15116)	6738 (5520-7671)	3576 (2891-4116)
MAITHON RB TPP	3585 (3143-3880)	1755 (1188-2172)	5702 (4216-6842)	1313 (952-1588)
MARWA TPS	3142 (2727-3445)	603 (479-699)	5404 (4446-6109)	842 (690-954)
MAUDA TPS	3260 (2830-3572)	3254 (2433-3886)	846 (653-989)	688 (518-819)

Table S9

Comparison of Mortality and Crop Damage Intensity (3/4)

Power Station	Mortality Damage Intensity ($/GWh-year)	Wheat Damage Intensity ($/GWh-winter)	Rice Damage Intensity ($/GWh-monsoon)	Avg Crop Damage Intensity ($/GWh-year)
MEJA STPP	3294 (2877-3772)	9719 (7605-11377)	3955 (3147-4558)	588 (467-680)
MEJIA TPS	4508 (3973-4834)	1019 (604-1348)	10160 (7941-11818)	1512 (1146-1789)
METTUR TPS	11514 (10092-12344)	12 (4-19)	4090 (3165-4779)	490 (378-573)
METTUR TPS II	15678 (13740-16811)	11 (2-17)	4085 (3167-4778)	669 (517-784)
MUNDRA UMTPP	7566 (6562-8323)	382 (272-474)	356 (244-437)	130 (91-160)
MUTHIARA TPP	1124 (985-1206)	0 (0-0)	3488 (2874-3939)	348 (287-393)
MUZAFFARPUR TPS	3814 (3346-4282)	6176 (4809-7267)	5795 (4701-6634)	1967 (1558-2289)
NABI NAGAR TPP	3954 (3453-4445)	6264 (4823-7374)	5746 (4588-6620)	2483 (1944-2893)
NASIK TPS	3877 (3367-4227)	1826 (1487-2096)	635 (521-719)	292 (238-334)
NAWAPARA TPP	2950 (2558-3218)	461 (383-522)	3857 (2963-4506)	322 (253-373)
NEYVELI EXT TPS	48083 (42149-51537)	0 (0-0)	5871 (4570-6903)	946 (737-1113)
NEYVELI TPS Z	44551 (39059-47738)	0 (0-0)	6715 (5366-7723)	1142 (912-1313)
NEYVELI TPS II	48646 (42649-52126)	0 (0-0)	6073 (4758-7089)	915 (717-1068)
NEYVELI TPS II EXP	44528 (39039-47711)	0 (0-0)	6021 (4696-7037)	972 (758-1136)
NIGRI TPP	3000 (2612-3343)	3832 (2812-4626)	1177 (882-1408)	769 (567-926)
NORTH CHENNAI TPS	4905 (4290-5279)	0 (0-0)	2233 (1773-2593)	286 (227-332)
OBRA TPS	3705 (3231-4152)	3390 (2355-4180)	1925 (1400-2325)	904 (638-1106)
PAINAMPURAM TPP	4244 (3699-4603)	0 (0-0)	1545 (1267-1755)	254 (208-288)
PANIPAT TPS	7174 (6259-8002)	13274 (10248-15568)	8290 (6732-9447)	4082 (3223-4728)
PARAS TPS	3727 (3231-4080)	2773 (1997-3363)	1028 (814-1190)	407 (298-489)
PARICHHA TPS	4159 (3614-4658)	9246 (7169-10842)	3298 (2609-3829)	2383 (1856-2788)
PARLI TPS	3639 (3161-3971)	2871 (2175-3398)	1057 (826-1227)	407 (315-475)
PATHADI TPP	3098 (2689-3388)	504 (393-590)	4711 (3888-5323)	710 (582-806)
PRAYAGRAJ TPP	3822 (3334-4349)	9117 (6982-10769)	3925 (3120-4530)	1891 (1466-2217)
RAGHUNATHPUR TPP	4532 (3985-4877)	1486 (975-1861)	7147 (5561-8364)	1221 (928-1444)
RAICHUR TPS	3982 (3457-4351)	476 (388-548)	2676 (2050-3144)	286 (223-334)
RAIKHEDA TPP	3545 (3074-3883)	784 (601-927)	6799 (5417-7819)	1256 (999-1447)
RAJIV GANDHI TPS	4087 (3568-4553)	14438 (11122-17021)	7796 (5951-9226)	3666 (2809-4332)
RAJPURA TPP	4420 (3851-4884)	15609 (12607-18017)	12297 (10285-13764)	4922 (4038-5604)
RAMAGUNDEM B TPS	3547 (3081-3872)	52 (6-85)	1661 (1270-1967)	151 (109-183)
RAMAGUNDEM STPS	3179 (2762-3468)	49 (7-81)	1696 (1307-1999)	254 (190-303)
RATIJA TPS	2380 (2058-2606)	451 (314-557)	4645 (3782-5279)	547 (436-629)
RAYALASEEMA TPS	5652 (4919-6147)	0 (0-0)	2786 (2192-3239)	357 (281-415)
RIHAND STPS	3205 (2790-3556)	2143 (1494-2627)	1287 (855-1614)	576 (395-712)
ROPAR TPS	2124 (1836-2353)	0 (0-0)	6556 (5213-7547)	1354 (1077-1559)
ROSA TPP Ph I	6890 (6029-7777)	12070 (9360-14159)	5639 (4481-6521)	1413 (1117-1640)

Table S9

Comparison of Mortality and Crop Damage Intensity (4/4)

Power Station	Mortality Damage Intensity ($/GWh-year)	Wheat Damage Intensity ($/GWh-winter)	Rice Damage Intensity ($/GWh-monsoon)	Avg Crop Damage Intensity ($/GWh-year)
SAGARDIGHI TPS	4503 (3962-4858)	1318 (956-1607)	9391 (7716-10668)	1665 (1351-1907)
SALAYA TPP	9220 (7991-10132)	0 (0-0)	878 (735-987)	253 (212-285)
SANJAY GANDHI TPS	2468 (2139-2713)	2586 (1872-3140)	1005 (763-1186)	654 (477-790)
SANTALDIH TPS	4239 (3716-4583)	1632 (1109-2021)	6265 (4801-7393)	1281 (960-1527)
SASAN UMTPP	2879 (2503-3181)	2385 (1724-2892)	1229 (819-1538)	594 (418-728)
SATPURA TPS	3439 (2975-3778)	4296 (3160-5132)	1182 (916-1374)	1080 (798-1289)
SEIONI TPP	3153 (2736-3472)	4692 (3621-5554)	1408 (1131-1628)	928 (721-1095)
SGPL TPP	4244 (3699-4603)	0 (0-0)	1507 (1231-1715)	257 (210-292)
SIKKA REP TPS	8794 (7621-9662)	667 (433-849)	804 (652-925)	190 (135-233)
SINGARENI TPP	2928 (2545-3192)	106 (41-157)	1365 (1013-1636)	209 (148-256)
SINGRAULI STPS	2741 (2385-3044)	2445 (1721-2984)	1239 (827-1554)	585 (405-721)
SIPAT STPS	3355 (2911-3678)	675 (524-789)	5955 (4953-6703)	823 (678-931)
SOUTHERN REPL TPS	18914 (16702-20217)	505 (342-631)	7585 (5874-8862)	1585 (1224-1854)
SURATGARH TPS	2885 (2502-3201)	3340 (1975-4350)	-21 (-323-259)	761 (404-1033)
SURAT LIG TPS	43267 (37523-47540)	2384 (1924-2759)	744 (612-848)	525 (425-606)
SVPL TPP	2066 (1787-2261)	0 (0-0)	4439 (3580-5069)	812 (655-927)
TALCHER STPS	2976 (2591-3232)	0 (0-0)	1797 (1332-2160)	210 (156-252)
TALWANDI SABO TPP	4542 (3966-5016)	16696 (12926-19626)	11072 (8620-12975)	4229 (3282-4965)
TAMNAR TPP	3836 (3329-4207)	263 (180-325)	3422 (2622-4047)	602 (456-715)
TANDA TPS	5193 (4565-5953)	10771 (8178-12727)	5004 (3916-5821)	3933 (3018-4622)
TENUGHAT TPS	3080 (2688-3356)	2320 (1746-2760)	3577 (2652-4284)	873 (650-1044)
TIRORA TPS	3293 (2859-3616)	3342 (2613-3914)	861 (612-1055)	661 (510-779)
TORANGALLU TPS SBU I	3629 (3149-3969)	326 (244-390)	2834 (2229-3281)	518 (405-602)
TROMBAY TPS	6956 (6044-7579)	200 (125-260)	130 (106-149)	49 (35-61)
TUTICORIN JV TPP	1211 (1061-1300)	0 (0-0)	1650 (1193-1984)	259 (187-311)
TUTICORIN TPS	1080 (946-1158)	0 (0-0)	2168 (1691-2510)	285 (222-330)
UCHPINDA TPP	2675 (2317-2922)	561 (471-635)	4937 (4025-5627)	723 (591-824)
UDUPI TPP	7483 (6505-8154)	1 (1-2)	6 (5-6)	0 (0-0)
UKAI TPS	5917 (5131-6487)	2166 (1701-2533)	678 (546-778)	469 (370-547)
UNCHAHAR TPS	4839 (4213-5536)	10703 (8194-12638)	5118 (4061-5927)	2572 (1992-3019)
UTRAULA TPS	3840 (3361-4289)	0 (0-0)	4767 (3909-5401)	983 (806-1113)
VALLUR TPP	6385 (5588-6868)	0 (0-0)	2399 (1926-2757)	249 (200-286)
VINDHYACHAL STPS	3111 (2707-3452)	2562 (1844-3105)	1238 (827-1551)	611 (430-748)
VIZAG TPP	2232 (1945-2422)	0 (0-0)	579 (480-658)	92 (76-104)
WANAKBORI TPS	4752 (4116-5228)	3685 (2778-4376)	1092 (878-1252)	846 (640-1003)
YAMUNA NAGAR TPS	6545 (5668-7316)	10510 (8278-12300)	7555 (6364-8476)	3294 (2653-3803)
